# Current Methods for Hyperpolarized [1-^13^C]pyruvate MRI Human Studies

**Published:** 2023-11-22

**Authors:** Peder EZ Larson, Jenna ML Bernard, James A Bankson, Nikolaj Bøgh, Robert A Bok, Albert P. Chen, Charles H Cunningham, Jeremy Gordon, Jan-Bernd Hövener, Christoffer Laustsen, Dirk Mayer, Mary A McLean, Franz Schilling, James Slater, Jean-Luc Vanderheyden, Cornelius von Morze, Daniel B Vigneron, Duan Xu, HP 13C MRI Consensus Group

**Affiliations:** 1 Department of Radiology and Biomedical Imaging, University of California, San Francisco, CA 94143, USA.; 2 UC Berkeley-UCSF Graduate Program in Bioengineering, University of California, San Francisco and University of California, Berkeley, CA 94143, USA.; 3 Department of Imaging Physics, MD Anderson Medical Center, Houston, TX, USA.; 4 The MR Research Center, Department of Clinical Medicine, Aarhus University, Aarhus, Denmark.; 5 GE Healthcare, Menlo Park, California, USA.; 6 Physical Sciences, Sunnybrook Research Institute, Toronto, Ontario, Canada.; 7 Department of Medical Biophysics, University of Toronto, Toronto, Ontario, Canada.; 8 Section Biomedical Imaging, Molecular Imaging North Competence Center (MOIN CC), Department of Radiology and Neuroradiology, University Medical Center Schleswig-Holstein (UKSH), Kiel University, Am Botanischen Garten 14, 24118, Kiel, Germany.; 9 Department of Diagnostic Radiology and Nuclear Medicine, University of Maryland School of Medicine, Baltimore, MD, USA.; 10 Greenebaum Cancer Center, University of Maryland School of Medicine, Baltimore, MD, USA; 11 Department of Radiology, University of Cambridge, Cambridge, United Kingdom.; 12 Cancer Research UK Cambridge Institute, University of Cambridge, Li Ka Shing Center, Cambridge, United Kingdom.; 13 Department of Nuclear Medicine, School of Medicine, Klinikum Rechts der Isar, Technical University of Munich, 81675 Munich, Germany.; 14 JLVMI Consulting LLC, Dousman, WI, USA; 15 Department of Radiology, Washington University, St. Louis, MO, USA.

**Keywords:** Hyperpolarized MRI, metabolic imaging, carbon-13, pyruvate, dissolution dynamic nuclear polarization

## Abstract

MRI with hyperpolarized (HP) ^13^C agents, also known as HP ^13^C MRI, can measure processes such as localized metabolism that is altered in numerous cancers, liver, heart, kidney diseases, and more. It has been translated into human studies during the past 10 years, with recent rapid growth in studies largely based on increasing availability of hyperpolarized agent preparation methods suitable for use in humans. This paper aims to capture the current successful practices for HP MRI human studies with [1-^13^C]pyruvate - by far the most commonly used agent, which sits at a key metabolic junction in glycolysis. The paper is divided into four major topic areas: (1) HP ^13^C-pyruvate preparation, (2) MRI system setup and calibrations, (3) data acquisition and image reconstruction, and (4) data analysis and quantification. In each area, we identified the key components for a successful study, summarized both published studies and current practices, and discuss evidence gaps, strengths, and limitations. This paper is the output of the “HP ^13^C MRI Consensus Group” as well as the ISMRM Hyperpolarized Media MR and Hyperpolarized Methods & Equipment study groups. It further aims to provide a comprehensive reference for future consensus building as the field continues to advance human studies with this metabolic imaging modality.

## Introduction

MRI with hyperpolarized ^13^C agents, also known as hyperpolarized (HP) ^13^C MRI, has shown great potential as a novel imaging modality, particularly for its ability to probe metabolic processes in real time. The first human studies with HP [1-^13^C]pyruvate were performed in 2011 in prostate cancer patients (1). Since then, there have been over 60 papers published with imaging results of human subjects from 13 different sites, with applications including prostate cancer, brain tumors, breast cancer, kidney cancer, pancreatic cancer, metastatic disease, liver disease, ischemic heart disease, diabetes and cardiomyopathies. The vast majority of these studies used [1-^13^C]pyruvate (1–63), where [2-^13^C]pyruvate (64) and ^13^C-urea (56) have been demonstrated too.

As clinical HP ^13^C MRI advances, there is a growing need to build consensus for best practices, which are critical for comparing data across sites, performing multi-site trials,deploying methods to new sites, partnering with vendors, and potentially for obtaining broader regulatory approvals. In March 2022, we initiated an effort to build consensus within the HP ^13^C MRI community with this opportunity in mind, and it was greeted with strong enthusiasm.

The “HP ^13^C MRI Consensus Group”, containing over 55 members from 27 sites, identified the area of greatest need and opportunity for consensus building to be HP [1-^13^C]pyruvate human studies, for the following reasons:

- Pyruvate is the most mature and widely used HP agent and has the most significant translational evidence emphasizing the potential clinical impact.
- Clinical trials, particularly multi-site trials, have the strongest need for consensus methods to ensure that data can be combined across sites.

**Figure 1:**
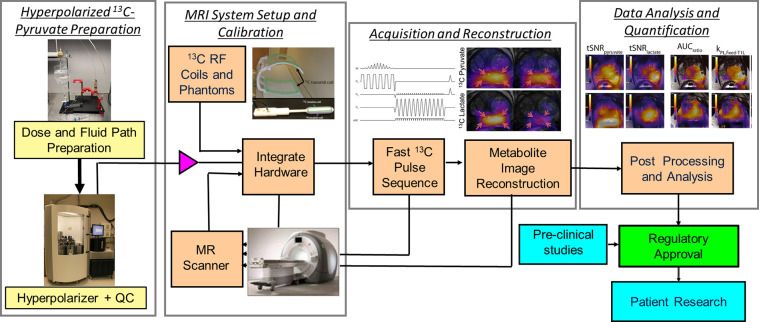
Illustration of the HP ^13^C MRI human study process, including the 4 major areas covered in this paper: Hyperpolarized ^13^C-pyruvate preparation, MRI system setup and calibration, Acquisition and Reconstruction, and Data Analysis and Quantification.

This work is a Position Paper for which the goal is to describe current successful practices and study methods for HP [1-^13^C]pyruvate human studies along with justification to support those practices. This is divided into four major topic areas: (1) HP ^13^C-pyruvate preparation, (2) MRI system setup and calibrations, (3) data acquisition and image reconstruction, and (4) data analysis and quantification (Fig. 1). The current successful practices and study methods include a literature review of published peer-reviewed journal papers showing human HP [1-^13^C]pyruvate study data, up to September 2022 (1–63), as well as new unpublished information from surveys of HP ^13^C study sites. Based on this information, we also highlight the evidence gaps, strengths, and limitations of current practices which are summarized at the end of each section.

### Hyperpolarized ^13^C-Pyruvate Preparation

This section covers the processes for creating the HP agent, ^13^C pyruvate, and will include many aspects and considerations that are needed to safely and effectively prepare doses for metabolic imaging studies in human subjects. These include material, personnel, equipment and facility, fluid path preparation, quality control, and release.

It is helpful to understand that the specifications of a dose of ^13^C pyruvate suitable for in vivo MR HP metabolic imaging were shaped in part by early preclinical studies performed by GE HealthCare summarized in Ref. (65). In short, the safety of the two novel drug components, ^13^C pyruvate and the electron paramagnetic agent (EPA) AH111501, were demonstrated in those studies. The more precise formulation of the dose suitable for human use was then determined from clinical studies (66) that included two Phase 1 clinical trials in young and elderly healthy volunteers without hyperpolarization of the ^13^C nuclei and another Phase 1/2a dose escalation and imaging feasibility study with HP ^13^C pyruvate in 31 prostate cancer patients at the University of California, San Francisco (UCSF) (1).

With the exception of the first HP ^13^C imaging clinical trial, which utilized a prototype device in a cleanroom (1), all HP ^13^C studies performed in humans to date have utilized the SPINlab polarizer (manufactured by GE HealthCare). Consequently all doses of the HP ^13^C pyruvate delivered by SPINlab have been produced using the “SPINlab Pharmacy Kit” that serves as the container-closure system for the various drug components (^13^C pyruvic acid and EPA mixture, dissolution medium, and neutralization and dilution medium) during sample polarization, dissolution and quality control (QC) processes. Thus many aspects of the HP sample preparation considerations discussed below are related to the SPINlab instrument and the consumables designed to be used with it (67).

### General considerations

While more than 860 patients or healthy subjects having been injected with HP ^13^C pyruvate as of January 2022 without reports of any serious adverse events (68), HP ^13^C pyruvate injection remains an investigational MR contrast agent and can only be administered by those with Investigational New Drug (IND) exemption from the Food and Drug Administration (FDA) in the USA, a Clinical Trial Application (CTA) in Canada, approval from National Research Ethics Committee Services in the UK, or approval from the relevant local regulatory body. Thus, methods and processes involved to produce a dose should have patient safety as the first priority. Since utilizing dissolution dynamic nuclear polarization (dissolution-DNP) for human use is still a relatively new development, there are no existing published regulatory guidelines specifically for this method.

There are two major production styles that determine how various sites approach the agent preparation. In the US, the most common approach is to rely on a sterilizing filter (“Terminal Sterilization”) to ensure sterility of the final product, akin to PET tracer production, where a starting molecule with a radioisotope is processed using various other ingredients to make the final, desired and injectable contrast agent within a necessarily short amount of time (69). For these sites, sterilization of the components and accessories upstream of this filter are not required, although many of them were manufactured and tested following Good Manufacturing Practice (GMP) or Good Laboratory Practice (GLP) requirements. The filling process is usually performed under an ISO 5 laminar flow hood, but a clean room or an isolator is not required. This approach is typically accompanied by testing the integrity of the sterilizing filter prior to release of the dose for injection. Typically, post release endotoxin and sterility tests are performed using an aliquot reserved from each released dose.

In the UK and EU, the most common approach is to more-closely follow sterile pharmaceutical compounding guidelines (70), where all components and ingredients are required to be sterile or manufactured under GMP guidelines and are assembled and filled within a clean room environment or an isolator system (“Sterile Preparation”). Typically a batch of Pharmacy Kits for HP ^13^C pyruvate injection are prepared together. The sterility of the final dose is also ensured by batch validation testing, in addition to the sterility of the ingredients and the sterile compounding process. The endotoxin and sterility testing are performed for the process validation but are not performed for each injected dose. Some institutions fill and assemble the Pharmacy Kit required for a specific study on the same day or the day prior to polarization, dissolution, and patient administration, but others have also demonstrated the feasibility of preparing a batch of kits, keeping them in a −20°C freezer and using them over a period of a few months.

Beyond the obvious requirements that the process and the facility has to ultimately produce a dose that is safe to inject into a human, regulatory authorities will also focus on the question “Are you in control of your processes?”. To be in control of your process requires an in-depth and broad understanding of all processes involved in pre, post, and during the production process.

### Personnel

It is typical and may be required to have licensed personnel involved in the production process depending on local regulations.Typically a pharmacist, radiopharmacist or other similarly qualified person (QP), in charge of the facility where the Pharmacy Kit filling and preparation is taking place, is responsible for the overall process and the release of the injectable dose. Qualified cleanroom technicians are often involved in the Pharmacy Kit filling under the supervision of the pharmacist or QP. As is required for pharmaceutical compounding or PET tracer production, training requirements and training records for all personnel need to be maintained and available for audit by the FDA or equivalent.

### Equipment and facility

The facility and all equipment need to have standard operating procedures (SOPs) that describe how equipment is used, maintained, and calibrated to comply with relevant legislation. Currently, almost all the filling of the Pharmacy Kit takes place within a compounding laminar flow hood or isolator (typically ISO 5). At some sites, the filling is conducted within a cleanroom, while at others, it is conducted in a dedicated non-cleanroom space, reflecting differences in cleanroom approach and specifications between regulators worldwide (71). Some equipment or facilities, such as the compounding hood or cleanroom, may require external certified laboratories for testing.

### Material handling

Material handling guidelines (69,70) require SOPs detailing a system to track all of the materials involved in the HP production process for a particular patient dose, similar to current good manufacturing practice (cGMP) requirements for material handling for drug compounding. This includes acceptance standards, storage conditions, amount used in the patient dose for each ingredient and materials used in the assembly of the fluid path and Pharmacy Kit. Currently some users choose to open and inspect and sometimes modify the Pharmacy Kits upon arrival, but some users keep them in the sealed packaging until they are required for dose preparation.

### Pharmacy Kit filling and assembling

As required by an IND or its equivalent, the preparation of the doses of HP ^13^C agent are detailed in the Chemistry, Manufacturing, and Control (CMC) section of an applicable regulatory submission; an example of this has been made available (72). It describes the processes of filling the Pharmacy Kit with the different components that make up the final drug product, and of assembling the final kit for either storage or immediate use in the polarizer. Special attention should be given to the laser welding process in order to satisfy installation qualification (IQ) and operational qualification (OQ). Typically, the final developed process is validated by process qualification (PQ) runs, during which 3 or more Pharmacy Kits are filled and used and the final HP ^13^C products are tested for endotoxin and sterility and to confirm that they meet the dose specifications for injections (usually including pyruvate concentration, residual EPA concentration, pH, liquid state polarization level and dose temperature). The data from 3 consecutive PQ runs are submitted as part of the IND submission (or its equivalent), and are often also reviewed by the Institutional Review Board (IRB) where the studies are conducted.

### Quality control and dose release

The quality control (QC) and dose release can be separated into two aspects: one is the QC and release of the filled Pharmacy Kit, and second is the QC and release of the HP ^13^C agent for injection, after polarization and dissolution. For institutions filling a batch of kits and storing them to use over a period of time, typically the batch can be released based on initial validation, environmental monitoring data from the day of kit production, and if filters are used during preparation of any of the components, filter integrity testing. But in some cases one or more kits are used for validation before the batch of kits are released for future use. For institutions that fill only the kits required for specific studies shortly before the experiment, the filled kits often do not go through separate release tests before they are used.

The quality control of the HP ^13^C pyruvate solution post dissolution is primarily performed to ensure that the agent meets the dose specifications (Table 1) before it is administered to the subject. These specifications target both safety (pH, residual EPA, temperature) and efficacy (pyruvate concentration, polarization, volume). Typically, the pyruvate concentration, residual EPA concentration, pH, dose temperature, dose volume, and liquid state polarization are measured by the QC accessory associated with the SPINlab polarizer. Some users perform a secondary measurement for one of the parameters, such as pH, using a different instrument or pH paper. For sites that do not go through a separate release testing process for batch filled kits, the integrity of the sterilization assurance filter, a part of the Pharmacy Kit, is typically tested as a part of the dose release. It is also common for these users to preserve an aliquot of the final HP ^13^C pyruvate solution for post-release endotoxin and sterility testing. This testing cannot be completed fast enough to test an individual dose prior to injection, but this is why other processes such as PQ runs and validation testing are done to minimize the chance a subject could be injected with a contaminated dose. The final dose release and injection should be done under the supervision of a licensed professional, based on local regulations.

### Some key challenges

Many of the challenges associated with HP ^13^C pyruvate preparation can be attributed to the conditions required for the dissolution-DNP method of high magnetic field (~3–7 T) and very low temperature (~1 K) during polarization, with pressurized and superheated water necessary for the rapid dissolution event. These extreme conditions are quite challenging for the design of the container-closure and fluid path system. In particular, the cryogenic temperature in the polarizer requires special attention to any moisture or ambient (moist) air introduced into that portion of the fluid path, which can form an ice block at ~1 K. This ice can lead to flow restriction during the dissolution event and reduce the strength of the laser welded bond between the cryovial and its cap. This can ultimately produce failures in the dissolution step, including variations in final pyruvate concentration and pH that may fail to meet QC release criteria as well as fluid path ruptures that provide no available dose and result in polarizer down-time.

The polarization of the HP ^13^C pyruvate sample decays quickly over the span of a few minutes after dissolution, and thus the process of dissolution, QC for release, and injection should be completed as fast as possible to preserve the high polarization level achieved. Any delays in the preparation process, such as transportation time or equipment malfunction, can significantly reduce the final polarization and result in lower quality imaging data.

### Current Practices

**Figure 3:**
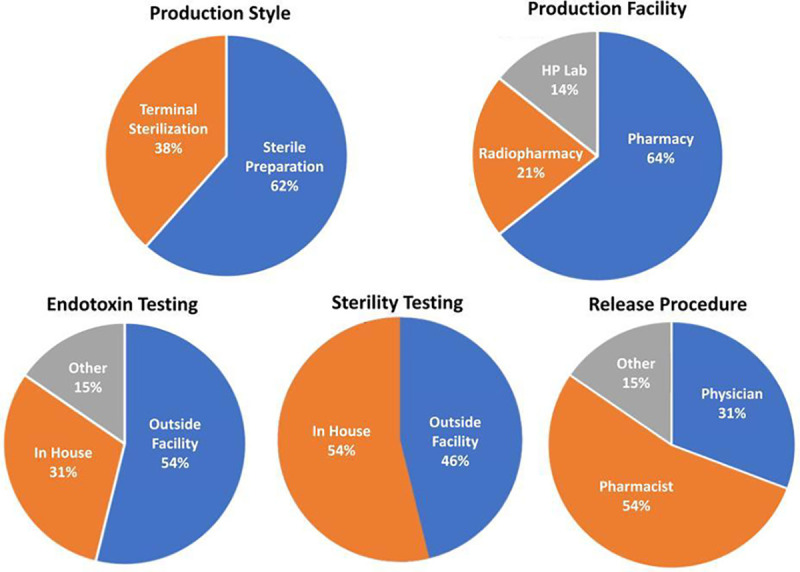
Hyperpolarized agent preparation methods reported by sites currently performing HP ^13^C MRI human studies

A summary of data collected from all sites performing clinical trials with HP ^13^C-pyruvate is shown in Fig. 3 and Table 1, including the specification of the final dose and how the quality control and release of the final dose are performed. There is a split in the Production Style, described in the General Considerations section above, with 8/13 sites using Sterile Preparation versus 5/13 using Terminal Sterilization. While many of the dose specifications show notable differences in acceptable ranges, all of these variations listed in tables have been successfully and safely been used to perform HP ^13^C pyruvate studies in humans. Their differences depend on the institutions’ preferences, resources and their particular regulatory situation. There is high similarity in pyruvate ranges, temperature ranges, EPA limits, and volume limits. There is modest variability in pH ranges and large variability in the endotoxin test limit. There is a 3-fold difference in acceptable polarization levels, which are measured to ensure a futile dose is not injected since the polarization is directly proportional to SNR. This reflects the decision by several sites to believe that useful data can be still be obtained with suboptimal polarizations.

### Summary

The overall safety record of HP ^13^C-pyruvate has been very strong, and the SPINlab hyperpolarizer has proven to provide high polarizations at human sized doses while meeting numerous QC and release criteria. A weakness remains the failure modes of the SPINlab Phamacy Kits (e.g. ice blocks, path ruptures), which are placed under extreme requirements particularly during dissolution. The preparation process still requires a high degree of expertise. Therefore, there is a significant need to improve the reliability, robustness, and ease of operation for generating HP ^13^C-pyruvate doses for human studies. Furthermore, there is a divide between manufacturing and sterile compounding style preparation as well as other site-specific practices, resulting in variations in SOPs and justification required to relevant regulatory bodies. There have also been no comparisons between these approaches. It is also unclear what release criteria and QC parameters are truly required to ensure patient safety. However, all of the reported methods are acceptable and approved by the appropriate regulatory authorities, and have led to the rapid expansion of successful human studies in recent years.

### MRI System Setup and Calibrations

This section covers the MRI system setup, including the imaging system, RF coils, phantoms, and prescan calibration methods.

### Imaging system

The main prerequisite for a given MRI scanner to be capable of supporting studies with HP ^13^C is its “broadband” capability to transmit and receive radiofrequency (RF) signal at the frequency of ^13^C, which is around 4 times lower than ^1^H. This does not come as a default on clinical MR devices. The transmit power of the broadband amplifier should also be sufficient to support the intended flip angle and RF pulse shape with the employed transmission RF coil(s) for ^13^C. Most studies to date use relatively low flip angles (< 90 degrees) for HP ^13^C in order to preserve polarization for time-resolved imaging. The capability to receive ^13^C signal on multiple channels is also desirable to increase SNR, as discussed further in the “RF coils” section.

The choice of magnetic field strength is primarily dependent on the metabolites’ frequency separation due to chemical shift dispersion and ^1^H imaging. High field strengths do not enhance hyperpolarized ^13^C signal as they do for ^1^H because the signal strength in a HP experiment relies on manipulating the population of quantum energy states outside of the MRI scanner. However, the injected HP ^13^C-pyruvate and its metabolic products have greater frequency separation at higher fields, and it may thus be easier to separate and quantify these resonances at higher fields. This comes at the cost of a reduction in the achievable T_2_* and often reduced T_1_. As the initial polarization is independent of the imaging field strength it has been proposed that the increased T_2_* at 1.5T can potentially be exploited to increase SNR by adapting the acquisition bandwidth or reduce off-resonance imaging effects in cases when the decay of the transverse magnetization is dominated by T_2_* (73). In practice, 3T has been used in all published human ^13^C-pyruvate studies surveyed (Supporting Table S1), and comprises the majority of scanners currently in use for human studies (Table 3). A field strength of 3T is well-suited for ^1^H MRI anatomical reference and correlative imaging.

Stronger and more rapidly slewing magnetic field gradients support more rapid spatial encoding, particularly for metabolite-specific single-shot imaging using echo-planar imaging (EPI) or spiral imaging (See “Acquisition and Reconstruction”). Although the spatial resolution acquired for HP ^13^C imaging is typically much coarser than for ^1^H MRI, the factor of ~4 in gyromagnetic ratio leads to the same reduction factor in performance of the gradient system, so ^13^C experiments are potentially more limited by gradient hardware performance. To date, all human studies have used the commercially-available integrated gradient systems provided in clinical MRI scanners.

Optimization of scanner design has understandably focused on minimization of artifacts in ^1^H MRI, where devices such as room lights, the gradient amplifiers, and the motors driving the patient bed are checked to ensure that they do not produce RF interference at the ^1^H frequency, but artifacts may arise at other frequencies. Eddy current compensation is also not always appropriately adjusted for nuclei at other frequencies (74). In order to optimize for ^13^C, many sites have performed checks on phantoms for RF interference, gradient artifacts, and eddy currents (74), including the use of post-hoc gradient impulse response function characterisation and correction, and some vendors have fixed these issues as well.

### RF coils

For HP ^13^C imaging studies in humans, RF coils for both ^1^H and ^13^C nuclei are needed, with ^1^H MRI providing an anatomical reference for registration and optional additional multiparametric MRI readouts. At the Larmor frequency of ^13^C nuclei, the relative contributions from coil noise compared to sample noise increase compared to ^1^H (73,75), although sample noise still is likely the dominant contributor for human-sized coils at 32.1MHz - the resonance frequency of ^13^C nuclei at 3T.

**Figure 4:**
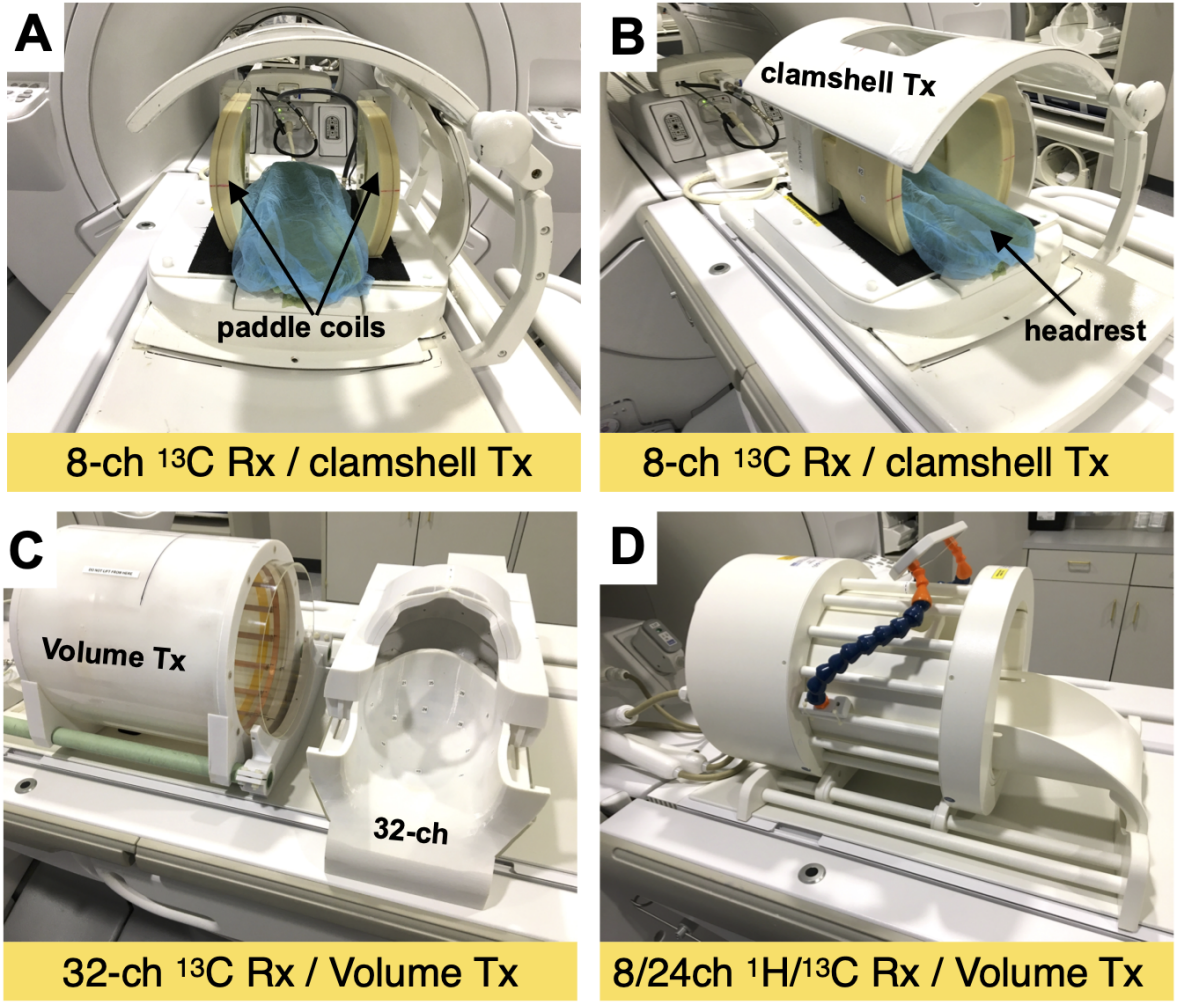
Examples of RF coil configurations used for human HP [1-^13^C]pyruvate brain studies. (A,B) ^13^C Clamshell TX (Helmholz pair) and 2× 4-channel paddle RX arrays. (C) ^13^C Birdcage volume TX and 32-channel RX array (RX array slides into TX coil). (D) ^13^C Birdcage volume TX and 24-channel RX array, combined with a ^1^H 8-channel RX array. Image reproduced with permission from Ref (16).

The key requirement for human ^13^C-pyruvate RF coils are that the coil geometry and sensitive volume must cover the volume of interest in the subject. Table 2 and Figure 4 shows coil configurations that have been used and optimized for applications in different anatomic regions.

Volume resonators are most commonly used for transmit, as they surround the subject to provide B1 transmit across the FOV (B_1_^+^). While ^1^H relies on a large birdcage (“body”) coil built into the scanner, ^13^C transmit coils must be placed inside the bore. This takes up valuable space within the magnet, and also has led to the use of designs with relatively inhomogeneous B_1_^+^. Many human studies have used Helmholz pair resonators for transmit, including the “clamshell coil”, which has a notably inhomogeneous B_1_^+^ profile but has been used because of relatively easy integration into the scanner bore. B_1_^+^ variation results in variations in the flip angles that control the use of the hyperpolarized magnetization and creates errors in common HP metrics (9,76). The exception are head coils, where birdcage designs with highly homogeneous B_1_^+^ can be placed around the head while easily fitting inside the bore.

As with ^1^H MRI, higher SNR can typically be achieved by smaller receive coil elements, such as surface coils or phased arrays, and the majority of ^13^C receive coils used have layouts similar to ^1^H phased arrays.

RF coil quality control is important to ensure proper functioning of the coils to provide consistent imaging quality, especially with limited natural abundance ^13^C signal *in vivo*. It typically involves 1) a physical integrity check of the coil cables and connectors and 2) phantom SNR tests to check the coil’s performance and to monitor it over time (see Phantoms below). An useful reference for RF coil quality control is outlined in the MRI accreditation program of the American College of Radiology (77) and can be adapted for ^13^C coils.

Notably, configurations for brain and prostate studies used dual-tuned ^1^H/^13^C coil designs, which greatly simplify workflow and registration of ^1^H and ^13^C images, as no switching of coils is needed.

### Phantoms

Since hyperpolarized magnetization is non-renewable, phantoms containing ^13^C nuclei are important to: 1) test the multi-nuclear capabilities of the imaging system, including all parts of the signal excitation and receive chain; 2) perform calibration measurements before a scan with hyperpolarized nuclei; and 3) perform necessary pre-scan adjustments (see “Prescan Calibration” section). The phantoms currently in use are listed in Table 3. Their composition must provide sufficient ^13^C signal, with additional considerations of conductivity, stability, chemical shift(s) present, potential for dynamic imaging, and cost. The phantom geometries are typically either compact, in order to be used alongside the subject during a HP scan, or large enough to mimic the inner volume of a RF coil for system testing.

One popular compact design contains enriched ^13^C-urea at high concentration, typically 8 M, which provides a single resonance, placed inside a small container ~1 mL. The most common recipe mixes ^13^C-urea in a 90% water/10% glycerol solution, with glycerol used to increase the urea solubility and doping with a Gd-based contrast agent to shorten T_1_ which increases the potential SNR per unit time. For example, when Dotarem is added at a 3:1000 volume ratio the ^13^C-urea T_1_ is around 500 ms and T2 is around 100 ms. However, when testing pulse sequences influenced by T_1_ and T_2_, doping should be used carefully. This phantom is suitable for frequency calibration, transmit gain calibration, sequence testing, and as a fiducial marker when placed next to a patient. However, enriched ^13^C-urea has a relatively high cost compared to natural abundance compounds.

For larger volumes (>100 ml), the phantoms most often used contain undiluted ethylene glycol, glycerol, or dimethyl silicone. These compounds have sufficiently high carbon concentrations to provide sufficient ^13^C signal even with the 1.1% natural abundance of ^13^C. These larger phantoms matching the inner volume of an RF coil are useful for coil testing, including transmit (B_1_^+^) and receive (B_1_^−^) coil profile mapping, as well as to mimic acquisitions using in vivo FOV requirements. In this case, size and conductivity should match the expected subject size in order to mimic coil loading and get a realistic estimation of B_1_+. Large-volume natural abundance urea phantoms have also been used by some sites, but suffer from higher conductivity compared to biological tissues. Typically, it is easier to increase the conductivity and hence coil loading of the non-conductive phantom by adding NaCl to match physiological loading (16,78).

Dynamic phantoms that aim to mimic metabolite kinetics have also been developed (79–81), and have the potential to more closely mimic the HP experiment, but so far these are not widely used.

### Prescan Calibration

Prior to performing an MRI acquisition, the so-called prescan procedure is used to set the shim parameters to maximize B_0_ homogeneity over the field of view (FOV) or a specific region of interest (ROI), the scanner center frequency (CF), the RF transmit gain, and the receiver gain. While this calibration procedure is usually automated for ^1^H, the lack of sufficient natural abundance ^13^C signal prevents use of automated methods. (Although natural abundance ^13^C lipid signal has been detected, there are so far no reports on using this signal for prescan.) Table 3 shows current practices across sites.

Maximizing B_0_ homogeneity is independent of the nucleus and is therefore performed prior to ^13^C imaging using the ^1^H water signal and existing shimming tools, such as by a standard automated process (“Auto Shimming”) or using high order shimming routines. Similarly, the ^13^C CF can be calculated from the ^1^H CF using a predetermined scaling factor that depends on the target chemical shift (82). Another common approach used is to have a small, high-concentration ^13^C phantom, e.g. 8M ^13^C-urea, integrated in the RF coil or placed next to the scan subject (1). The reference frequency can also be based on real-time measurements after the HP injection but prior to imaging (83). Both the CF and B_0_ shimming are critical when using spectrally-selective RF pulses, as inmetabolite-specific imaging methods, where the desired excitation bandwidths are typically very narrow and frequency offsets can lead to a failure mode that is only apparent after injection.

The calibration of the RF transmit power is typically performed on a small, high-concentration ^13^C phantom placed near the region of interest during the scan or on a large ^13^C phantom of similar size and coil loading as the subject, prior to the subject scan. Reference power is often done by sweeping the power in a pulse-acquire sequence (53,62), or the Bloch-Siegert method (52,84). When using a small phantom, the location of the phantom, B_1_^+^ inhomogeneity as well as any shielding effects, e.g., when the phantom is integrated into a coil (1), may degrade the accuracy. Other methods include real-time Bloch-Siegert method measurements after the HP injection (83), and using the stronger natural abundance ^23^Na signal that is close enough to the ^13^C resonance frequency to be detected by ^13^C coils (82).

The receiver gain is predetermined, either systematically based on independent phantom measurements and assuming the dose and polarization of the HP compound is known prior to injection, or based on past HP imaging studies.

## Summary

Commercially available 3T MRI systems are by far the most commonly used for human HP ^13^C-pyruvate studies, although a systematic investigation of the impact of B_0_ has only recently been investigated (73). The multi-nuclear RF transmit and receive chain has proven sufficient for current acquisition strategies, although many sites have observed artifacts due to RF interference, gradient interference, and residual eddy currents when operating at the ^13^C frequency. A variety of ^13^C RF coils, tailored for numerous anatomical targets, have been successfully demonstrated, with the main limitation that most transmit coils take up a lot of additional space inside the bore and provide relatively inhomogeneous B_1_^+^ profiles. The phantoms used have converged into generally 2 categories - small phantoms containing ^13^C-enriched compounds that can be used during the study and human-sized phantoms containing compounds with high carbon concentrations but without ^13^C enrichment that are used to test and calibrate the coils. There are no standardized compositions or geometry, and dynamic phantoms that recapitulate in vivo kinetics would be desirable but are still an emerging area. Prescan calibration procedures were not well defined in most publications, so we surveyed individual sites to determine current practices. Calibration procedures for the B_0_ field (^13^C CF and shimming) for most sites take advantage of ^1^H signal and methods, while methods for calibration of B_1_^+^ is more variable across sites, likely a reflection of remaining challenges in how to perform this calibration. Standardization of both phantoms and calibration procedures would synergistically improve the robustness and reproducibility of HP ^13^C studies.

### Acquisition and Reconstruction

Data acquisition strategies in human HP [1-^13^C]pyruvate MRI studies must account for multiple chemical shifts, efficiently utilize the non-renewable HP magnetization, and acquire data quickly relative to metabolism and relaxation decay processes. These studies require spectral encoding to separate metabolites, necessitating pulse sequences that efficiently encode up to 5D data (3 spatial + 1 spectral + 1 temporal dimension). RF pulses must efficiently sample without immediately saturating the non-renewable HP magnetization, and sequences must acquire data quickly and be robust to both experimental and physiologic variation (e.g. B_1_^+^ inhomogeneity, variation in perfusion) to ensure reproducibility and minimize scan-to-scan variability. This section covers current successful practices for data acquisition in human [1-^13^C]pyruvate studies, and accompanying ^1^H imaging, from different anatomic regions, including scan parameters and image reconstruction.

#### Acquisition and Reconstruction Methods

**Figure 5:**
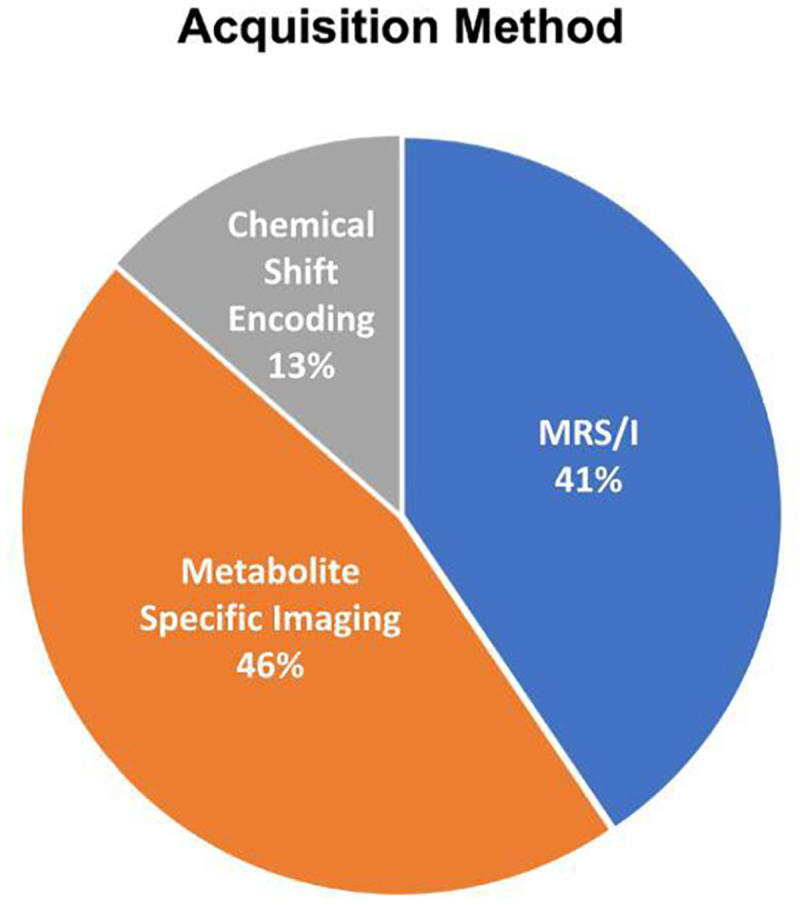
Acquisition methods used in published HP [1-^13^C]pyruvate human studies published up to September 2022, classified into: MR spectroscopy and spectroscopy imaging (MRS/I); chemical shift encoding methods, such as IDEAL, that use multiple TEs and model-based reconstructions; and metabolite-specific imaging methods that use spectrally-selective excitation to image a single resonance at a time.

The acquisition methods used in human [1-^13^C]pyruvate studies can be classified into 3 categories: 1) MR spectroscopy or MR spectroscopic imaging (“MRS/I”), 2) chemical shift encoding methods, and 3) metabolite-specific imaging (Fig. 5). MRS/I methods specifically resolve a spectrum that can be analyzed to extract expected as well as unexpected resonances, making this approach very robust. It was used in many initial studies (1). Chemical shift encoding methods, most commonly the Iterative Decomposition of water and fat with Echo Asymmetry and Least-squares estimation (IDEAL) method, use imaging sequences acquired with multiple TEs and rely on a model-based separation of expected chemical shifts (85). Metabolite-specific imaging methods use specialized RF pulses that are spatially and spectrally selective to excite individual metabolites which are then typically imaged with fast k-space trajectories such as echo planar imaging (EPI) or spirals (86). Their application to different organ systems is described below.

The image reconstruction methods used in human [1-^13^C]pyruvate studies have typically been conventional methods (e.g. FFT, non-uniform FFT, or equivalent). The incorporation of accelerated imaging and advanced reconstruction methods including parallel imaging (4,57,87) and compressed sensing (7) has also been applied in human studies for improved spatial resolution, temporal resolution and coverage, but have the potential for additional artifacts as well as SNR losses due to ill-conditioning of the reconstruction (e.g. g-factor). The majority of published studies do not use accelerated imaging indicating the resolution and coverage achievable without acceleration is currently adequate for successful data collection. Performing coil combination, even with fully sampled data has also been shown to have specific challenges for HP human images: using naive sum-of-squares methods suffer from high noise amplification in the relatively low SNR regime of HP [1-^13^C]pyruvate (compared to ^1^H), motivating several HP ^13^C-specific methods that include data-driven coil sensitivity estimation which have shown obvious improvements over sum-of-squares (11).

More recently denoising techniques have been applied as post-processing of human HP data(41,42,44). The techniques applied are based on spatial-temporal singular value decomposition for unsupervised estimation of signal and noise components. They have shown improvements in apparent SNR in the brain and liver, while care must be taken to choose parameters such as the rank threshold to avoid oversmoothing and overfitting to the estimated signal components.

#### Prostate Studies

Prostate cancer was the first human application of HP [1-^13^C]pyruvate (1), and data was acquired with MRS/I methods: 1D dynamic MRS, single-slice 2D dynamic echo-planar spectroscopic imaging (EPSI), and single time point 3D EPSI. Advances in imaging strategies led to the development and application of new acquisition schemes, including undersampled 3D EPSI with compressed-sensing (7), model-based chemical shift encoding methods that use *a priori* information (47,59), and metabolite-specific EPI (10), all of which can provide volumetric whole-organ coverage and dynamic acquisitions.

The pyruvate bolus arrival in the prostate can vary by ± 10 s between patients, necessitating dynamic imaging to reliably and consistently capture the pyruvate bolus (18). For this reason, all currently ongoing studies acquire dynamic data. While MRS/I, chemical shift encoding, and metabolite-specific imaging can all achieve dynamic imaging, chemical shift encoding and metabolite-specific imaging provide greater dynamic and volumetric coverage (85). For scan prescriptions, the FOV is designed to provide full prostate coverage and typically to match the orientation of the anatomic imaging used for registration. Flip angles used in current studies are constant through time, as quantification with a variable-through-time flip scheme is highly sensitive to bolus timing (8) and errors in the RF transmit (B_1_^+^) field (76).

#### Heart Studies

Data acquisition methods for ^13^C imaging in the heart must be designed to meet the demands of significant cardiac motion and blood flow. To cope with the periodic cardiac motion, most human heart studies to date used gating to the diastolic window, the longest cardiac cycle interval, which has reduced motion (2,22,28,30,35,36,38,45,52). The duration of the diastolic window limits the available data sampling time, making cardiac acquisitions the most time-constrained of the HP ^13^C MRI applications. The most common acquisition approach is metabolite-specific imaging with spiral k-space trajectories (2). Their single-shot imaging capability makes these methods particularly robust to motion effects. Furthermore, spiral k-space trajectories provide rapid k-space coverage and relatively benign flow and motion artifacts. The majority of studies have used 2D multi-slice acquisitions, but 3D encoding has also been used successfully (35).

#### Brain Studies

For HP ^13^C MRI of the human brain, the majority of studies have also used 2D (slice selective) acquisitions (10–12,14,16,28,33,40,41,44,51,53,60), with a trend toward volumetric coverage using 2D multi-slice metabolite-specific imaging. 3D metabolite-specific imaging of the whole brain, with phase encoding of the slice direction (34,57), has been shown to provide similar SNR efficiency (88) compared with multislice imaging. A number of studies have employed MRS/I (5,6,29,31–33,50,55) resulting in a spectrum from each voxel, which has the advantage of not requiring a priori information about which peaks to encode. This was important in early brain studies when it was not known which peaks would be detectable. Chemical shift encoding, using a set of images with different echo times and an iterative reconstruction of the individual resonances (i.e. the IDEAL approach (85)), has also been used (12,49,54), with the drawback that coverage in the slice direction was limited due to the time required to acquire multiple echo time images.

#### Abdomen and Breast Studies

The fundamental approaches to data acquisition and reconstruction in the abdomen and breast are largely similar to the aforementioned applications, but demand attention to particular challenges associated with these anatomic regions, especially relating to respiratory motion.

Although it has been shown that a basic 2D MRSI approach based on phase encoding and FID readout can be successfully applied for HP ^13^C imaging in breast (15) and kidney (13), major advantages in terms of spatiotemporal resolution and coverage have been realized using tailored approaches based on metabolite-specific imaging (43,62) and chemical shift encoding (43), which have facilitated multi-slice or 3D dynamic acquisitions over large FOVs in the abdomen (4,37,46).

The significant respiratory motion encountered in these regions can directly blur ^13^C images, and has further favored these rapid acquisition strategies. Motion also degrades B_0_ homogeneity, which can shift frequency-selective excitation profiles and introduce artifacts into rapid imaging readouts. This makes accurate determination of the acquisition center frequency and shimming essential in these regions which often cover large FOVs. (See “Prescan Calibration” section for more information). In some studies, breath-holding was used to minimize motion effects and enforce frame-to-frame data consistency (42). A pragmatic and reasonably effective approach for dealing with respiratory motion during ^13^C data acquisition is an initial breath-hold (as long as can be tolerated), followed by free-breathing (46,62).

#### ^1^H Imaging

Collection of ^1^H imaging data is essential both for prescribing the ^13^C acquisition and for interpretation of the resulting ^13^C data. Multi-planar ^1^H scouts are acquired prior to ^13^C acquisition to enable graphical prescription of the ^13^C imaging region. All human HP ^13^C-pyruvate imaging studies acquire conventional MRI scans (e.g. T_1_- and T_2_-weighted volumes) for anatomic reference, aiming to cover at least the full ^13^C FOV. Acquiring these anatomic scans as close as possible to the time of ^13^C imaging (immediately before or after) minimizes potential misregistration between the data sets. Depending on the application, other advanced ^1^H sequences are also acquired (e.g. diffusion-weighted imaging for cancer imaging). When contrast-enhanced data is acquired, it is done after ^13^C imaging, as paramagnetic contrast agents will accelerate ^13^C relaxation.

### Reported Study Parameters

**Figure 6:**
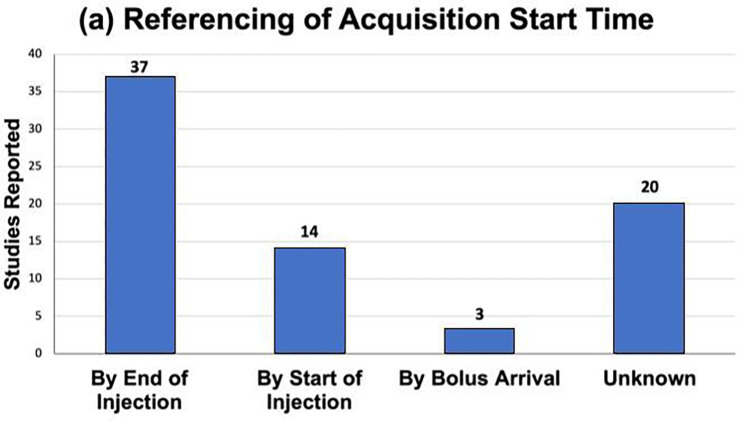

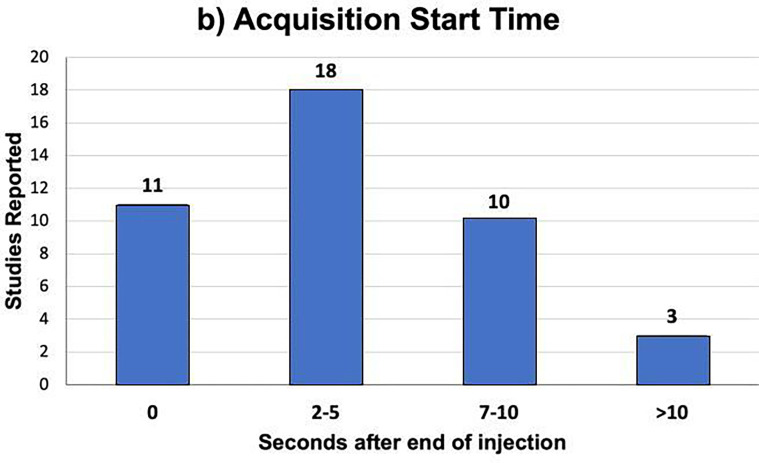

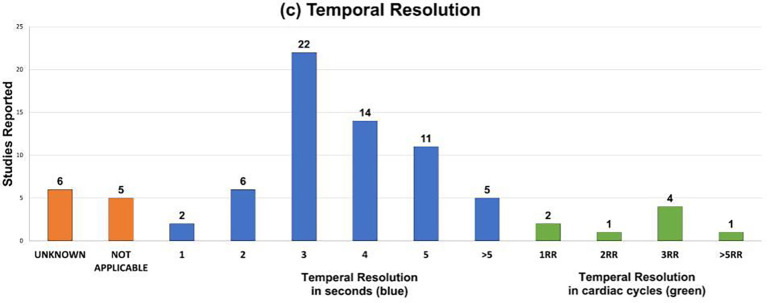
Temporal acquisition characteristics reported in HP [1-^13^C]pyruvate human studies published up to September 2022. (a) Reported referencing of acquisition start times. (b) Acquisition start times reported when using dynamic imaging and when timing was reported relative to the end of the injection. (c) Temporal resolutions. “Not Applicable” indicates dynamic imaging was not used.

Figures 5 and 6, and Supporting Table S2 shows the reported acquisition study parameters for human HP [1-^13^C]pyruvate studies published as of September 2022. Figure 5 shows a mixture of MRS/I, metabolite-specific imaging, and chemical shift encoding methods have been successfully used, where spectroscopy-based methods have become less prevalent in recent studies. Figure 6 shows the acquisition timing, including the important start time and interval/temporal resolution, is quite variable across studies.

### Summary

Three general categories of acquisition strategies have been used successfully for human HP ^13^C-pyruvate studies: MRS/I, model-based chemical shift encoding (e.g. IDEAL) methods, and metabolite-specific imaging methods. These have enabled successful studies in the prostate, heart, brain, abdomen, and breast. Recent studies increasingly have used the imaging-based strategies of metabolite-specific imaging and chemical shift encoding which are the fastest methods, although a heads-to–head comparison between techniques has not been performed. Metabolite-specific imaging is quite popular because of its speed and compatibility with single-shot imaging, but is sensitive to B_0_ field variations and thus requires careful calibrations. Nearly all studies surveyed acquired data dynamically, allowing measurement of the bolus and metabolite kinetics. The exact timings and associated flip angles vary quite widely across reported studies, with no consensus yet as to how to choose these parameters. Image reconstruction is typically done directly using Fourier Transform methods, and accelerated imaging strategies are uncommon.

### Data Analysis and Quantification

This section covers the analysis of data from human HP [1-^13^C]pyruvate studies, including modeling and metrics, visualization, as well as considerations for how to store data and metadata. Depending on study design, the analysis may need to give quantitative or semi-quantitative output reflecting a biological process or may just reflect a contrast between different regions of interest for quantitative evaluation.

#### Metrics

Metabolite images are commonly used as summary metrics for HP MRI data, often including some form of normalization as well as summed over time as an area under the time curve (AUC) (17). These are analogous to the visual evaluation that is most used for routine clinical work (89,90). In these metabolite images, we expect that the [1-^13^C]pyruvate AUC signal is predominantly weighted towards perfusion and uptake, while [1-^13^C]lactate, [1-^13^C]alanine and ^13^C-bicarbonate AUCs represent metabolic conversion. The strength of this approach lies in its simplicity and relatively few underlying assumptions. Limitations to the use of single-metabolite images or AUCs include sensitivity to inhomogeneous coil profiles (57,87,91), the acquisition strategy and acquisition parameters, pyruvate polarization and concentration level, and signal relaxation rates (92). Further, the reader must be careful to interpret all the images in conjunction to better understand the underlying biology; for example, increased [1-^13^C]lactate in the presence of decreased [1-^13^C]pyruvate delivery can have a very different meaning compared to increased [1-^13^C]lactate with increased [1-^13^C]pyruvate delivery.

In an attempt to address variations in coil sensitivity, polarization level, and pyruvate delivery, AUC images are often computed by normalizing to a specified parameter, such as the maximum pyruvate or average lactate signals, or presented as a ratio such as lactate/pyruvate or divided by “total Carbon” - the sum total of HP ^13^C signal observed across all metabolites. The AUC ratios between metabolites and pyruvate are proportional to the corresponding forward kinetic rates (81,93), but are not directly comparable to rate constants when magnetization loss rates (e.g. relaxation and losses due to signal excitation) differ between studies. Similarly, the ratios between the produced metabolites (e.g. bicarbonate/lactate) can reflect the balance between downstream metabolic pathways (12,55). Care must be taken to consider how AUC images are calculated and normalized before comparing values between studies.

To further quantify the interpretation, pharmacokinetic (PK) modeling approaches were developed to compute the apparent kinetics of pyruvate-to-metabolite exchange (92,94–99). These yield semi-quantitative to quantitative apparent rate constants, given in s^−1^. Some models require a vascular input function, while others avoid this requirement (95). PK models can explicitly account for acquisition-specific details such as excitation angle and repetition time, and thus may reduce the effects of these details on quantification. An input-less model, provided in the Hyperpolarized-MRI-Toolbox (https://github.com/LarsonLab/hyperpolarized-mri-toolbox) (100) and thus frequently employed for human data, has been shown to fit well and robustly to prostate and brain data (8,20). PK models are quantitative in nature, arguably provide more relevant biological information (8,20), and appear to be reproducible across sites (51). However, rate constants derived from PK models are still apparent rates, and likely do not reflect a single biological characteristic.

Some additional considerations include whether complex or magnitude data is used, as the noise behaviors will impact the analysis differently. Additionally, cut-off thresholds or other criteria may be used to identify and avoid voxels with insufficient SNR before analysis to improve robustness (20,41).

Regardless of the analysis approach, the underlying biology is not always clearly represented by the data; instead, the metrics may be influenced by perfusion, barrier permeability, intercellular shuttles, enzyme activities, co-substrate concentrations, or combinations thereof, depending on the organ and disease of interest (19,43,94,101–103). This may be addressed by incorporating complementary information. As an example, HP ^13^C pyruvate data is influenced by perfusion, and thus addition of perfusion MRI could be important for interpretation (98,104,105).

**Figure 8:**
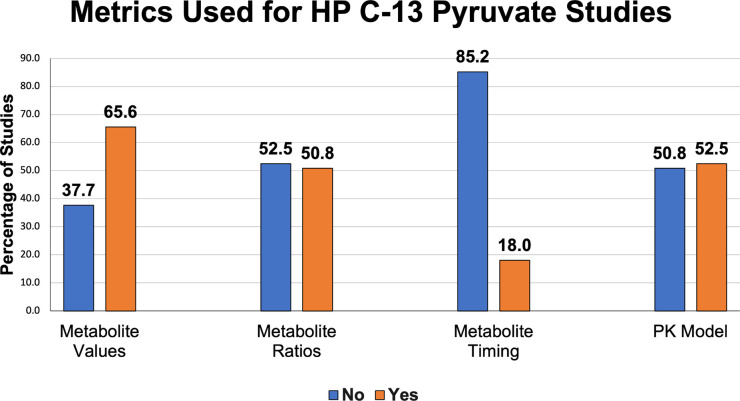
Reported metrics used for analysis in HP [1-^13^C]pyruvate human studies published up to September 2022.

All the methods outlined above have been explored in clinical studies, described in Supporting Table 3 and summarized in Figure 8. As of September 2022, approximately 52% of studies involving human subjects report rate constants derived from a PK model with a few different models reported. A nearly equal fraction (51%) of the studies report AUC ratio values. Approximately 66% of these studies report metabolite-specific images or AUC values. About 40% report SNR values; this metric is particularly frequent in manuscripts that describe technical developments for clinical HP MRI. Approximately 16% of these studies summarize model-free metrics, and 10% report measurements from a single timepoint. Most studies report a combination of quantities.

### Visualization

A wide variety of approaches have been used for visualizing data from human HP ^13^C-MRI studies. The challenges and practical considerations are: 1) choosing the appropriate metrics to display, 2) how to encode the parameters (e.g. the colormap), and 3) choosing how to provide anatomical context and other multi-parametric data. The choice of visualization also depends on the goal which could be for diagnostic interpretation, but also quality control, reproducibility among readers and publication.

#### Metrics

The choice of HP ^13^C metrics is described in detail above. At this stage in HP 13C development where there is no standardized metric, often a combination of metabolite images and ratios or PK model parameters are shown.

#### Parameter Encoding

**Figure 9:**
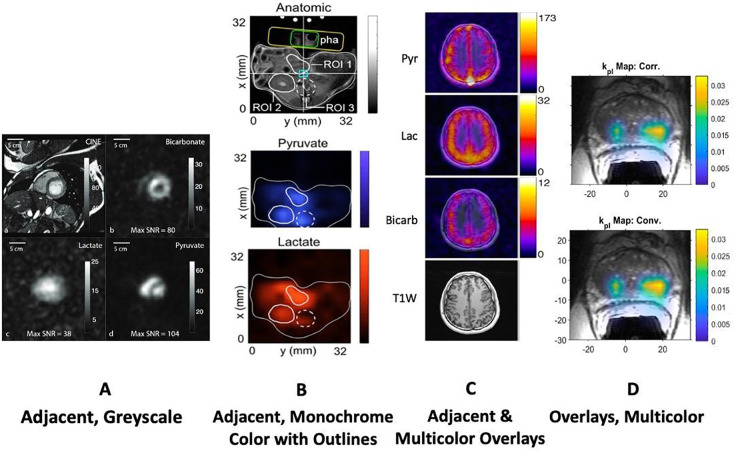
Examples of different parameters, color schemes and anatomical reference used to visualize HP ^13^C MRI: (A) cardiac study showing each metabolite (SNR) on one monochromatic grayscale image and separate anatomic reference, no overlays (2); (B) preclinical cardiac study showing one image per metabolite in monochrome (red / blue) on outlines of anatomy, and additional annotated anatomical image in separate panel (108); (C) brain study where each metabolite AUC (multicolor) was fused with an anatomic image, plus one anatomical image (28); (D) prostate study showing k_PL_ as multicolor overlay on anatomic image (26).

The mapping function chosen should provide an adequate, often quantitative, impression of the parameter mapped. There is a consensus in the visualization field that perceptually uniform maps are best suited to visualize continuous parameters, like the greyscale typically used by radiologists as well as other monochrome (black to blue) and color ranges (fire-type, rainbow-type) (106,107). Multi-color heatmaps have been the most frequently employed method for HP ^13^C data, while greyscale has infrequently been used but it ensures there is no coloring-based bias as well as facilitating later reuse (Fig. 9a). Among the color schemes employed in the clinical HP ^13^C literature, fire-type scheme seems to be the most common [similar to “Plasma” or “Inferno” in matplotlib.org]. Next most commonly employed is the rainbow-type scheme [similar to “Rainbow” in matplotlib.org].

#### Anatomical Context

HP MRI faces the challenge that it does not necessarily depict the anatomical features, similar to PET, and thus requires an anatomical reference. Most often, a grayscale anatomical image is overlaid with a HP colormap (Fig. 9c,d). This approach is very intuitive, but can skew perception as the grey-scale anatomical reference may affect the brightness of the HP data (e.g. signal in the skull). This bias does not occur when showing adjacent maps (Fig. 9a, b). Here, anatomical outlines may help to provide reference (Fig. 9b).

### Summary

Three general categories of analysis metrics have been reported: metabolite images (often normalized), metabolite ratios, and PK model parameters. There is no apparent convergence to one of the categories, and many studies report multiple metrics in their analysis. A standard metric that is robust to experimental variations (known or unknown) would be highly desirable to compare results across studies. PK modeling of conversion rates (k_PL_, k_PB_) have shown the most promise in this regard. HP ^13^C-pyruvate data has always been visualized with ^1^H anatomical reference images, allowing for localization of metabolism information. Most papers show HP data as color overlays, but using a variety of colormaps. Using a consistent, perceptually-uniform colormap would facilitate better translation of HP ^13^C-pyruvate MRI, and we can probably learn from clinical PET practice where metabolism data is also visualized alongside anatomical reference images.

## Discussion and Conclusions

Hyperpolarized [1-^13^C]pyruvate MRI has made great strides in recent years, with an explosion of human research studies covering a broader range of applications and performed by more sites worldwide. We have systematically surveyed the methods for HP agent preparation, system setup and calibration, acquisition and reconstruction, and data analysis used in previous and current studies. The results of this in this paper highlights that the areas of common procedures include the use of the same clinical hyperpolarizer, ^13^C phantoms configurations for testing and calibration, time-resolved imaging with a trend towards imaging-based acquisitions, and the use of area-under-curve and PK model based metrics. It also highlights that the areas of variations include the fluid path preparation approach and agent release criteria, system and experiment calibration procedures, choice of imaging-based acquisition and acquisition timing, and numerous metrics applied for analysis. See the summary for each section above for additional details.

In preparing this paper we also observed notable omissions in reporting of study methods that we believe are relevant to the study results. It was often difficult to determine the calibration procedures used. HP pyruvate preparation processes were often minimally described or left out entirely. In some studies, the key parameter of acquisition timings was not reported. Based on these findings, we believe it would be valuable to define guidelines for reporting of studies to ensure a minimum set of methods is described.

There is some potential bias in our findings and reported methods because one site (UCSF) has published more human HP [1-^13^C]pyruvate than other sites, accounting for 25 out of 63 of the papers reviewed. To mitigate this bias, we show the results of current practices submitted from different sites and have included named authors representing a variety of sites as well.

The next steps we propose are formal consensus building, in which a voting process will be used in the hopes of creating a concrete set of recommendations and best practices, as well as clearly identify where there is a lack of consensus and need for further development. It’s important to note that HP ^13^C-pyruvate MRI is still a research study, and therefore, the methods described and recommended will be treated as research from a regulatory point of view. With the development of guidelines and best practices, we hope to improve the reliability and reproducibility of results in future studies, and facilitate the translation of this technology to clinical applications.

## Supplementary Material

Supplement 1

## Figures and Tables

**Figure 2:**
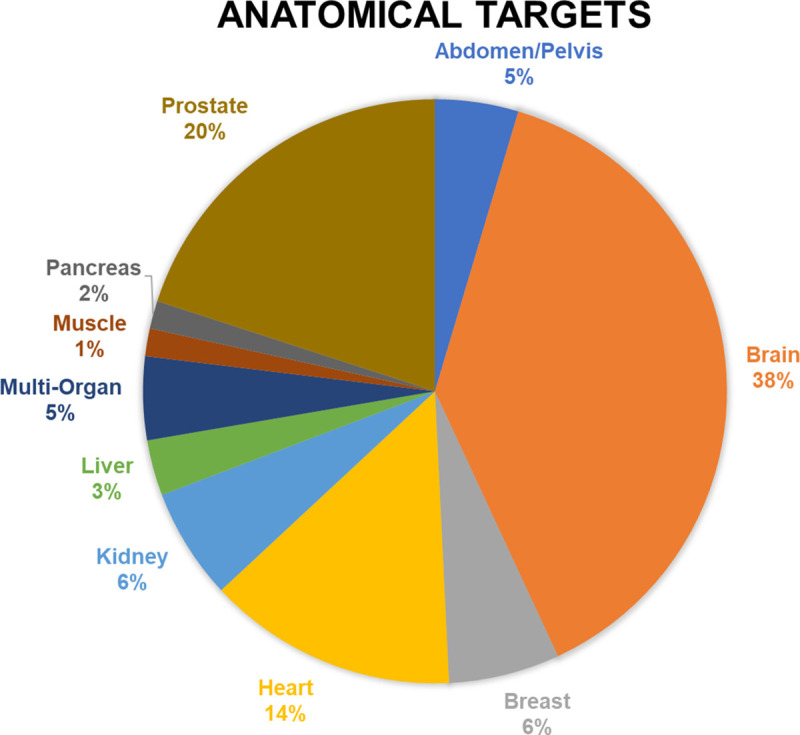
Anatomical targets of HP [1-^13^C]pyruvate MRI human studies published up to September 2022.

**Figure 7:**
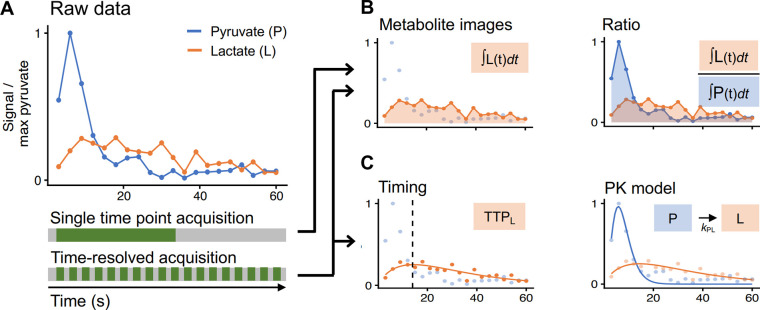
HP [1-^13^C]pyruvate raw data (A) have typically been quantified using four categories of metrics depending on the acquisition. Data acquired as a single time point are often quantified using normalized metabolite images or metabolite ratios (B). Dynamic data can be quantified using normalized metabolite images or metabolite ratios (B), or with metabolite timings such as time-to-peak (TTP) or pharmacokinetic (PK) models (C). The latter two require the data to be time-resolved. [1-^13^C]alanine and ^13^C-bicarbonate are analyzed similarly to [1-^13^C]lactate but omitted here for display.

**Table 1:** HP ^13^C-pyruvate preparation parameters, methods, and dose specifications used for quality control testing and release as well as validation. These were obtained from a survey of all sites performing clinical trials with HP [1–^13^C]pyruvate. The parameters used for product release are noted in **bold text**, otherwise these parameters are measured for batch validation or other QC measurements. The endotoxin and sterility testing are performed during process validation of the batch and/or post-injection, and largely depends on the agent production approach.

Site#	Production Style	Facility	Release Procedure	pH	Pyruvate [mM]	Residual EPA [mM]	Polarization, Liquid state [%]	Temperature [oC]	Volume [mL]	Appearance test performed	Filter Integrity Test	Endotoxin Test [EU/mL]	Endotoxin Testing	Sterility Test	Sterility Testing
1	Sterile Preparation	Pharmacy	Other	**4 – 9**	**200 – 300**	**<2.6**	**> 7.5**	**20–42**	**≥35**	Yes	**Yes**	<3.7	Outside	No growth	Outside
2	Sterile Preparation	Radiophar macy	Physician	**5 – 9**	**200 – 280**	**<5.0**	**>5.0**	**25–37**	**≥40**	**Yes**	No	<0.02	Outside	No growth	In House
3	Sterile Preparation	Pharmacy	Pharmacist	**6.7– 8.3**	N/A	**<3.0**	**≥15**	**25–40**	**>38**	**Yes**	No	<0.125	Outside	No growth	Outside
4	Terminal Sterilization	Hyperpola rized	Other	**6.5– 8.5**	**150 – 280**	**<3.0**	NA	25–40	6 ml + patient dose	NA	**Yes**	<5.0	Other	No growth	In House
5	Sterile Preparation	Pharmacy	Pharmacist	**6.7– 8.2**	**220 – 280**	**<3.0**	**≥5**	**25–39**	**>40**	**Yes**	No	<11	Outside	No growth	Outside
6	Sterile Preparation	Pharmacy	Pharmacist	**6.5–8.2**	**210 – 280**	**<3.0**	≥15	**25–39**	**>40**	**Yes**	**Yes**	<0.5	Outside	No growth	Outside
7	Terminal Sterilization	Pharmacy	Pharmacist	**6.5–8.5**	**200 – 280**	**<3.0**	**>15**	**25–40**	**≥38**	Yes	**Yes**	<4.37	In House	No growth	In House
8	Sterile Preparation	Pharmacy	Physician	**6.5–8.9**	**220 – 280**	**<3.0**	**≥15**	**25–37**	**≥40**	**Yes**	No	<0.5	Outside	No growth	Outside
9	Terminal Sterilization	Hyperpola rized	Pharmacist	**5 – 9**	**185 – 280**	**<5.0**	NA	25–40	**>38**	**Yes**	**Yes**	<2.5	In House	No growth	In House
10	Sterile Preparation	Pharmacy	Physician	**6.5–8.5**	**220 – 280**	**<3.0**	≥10	**25–37**	**>38**	**Yes**	No	<0.1	Outside	No growth	Outside
11	Terminal Sterilization	Pharmacy	Physician	**6.5–8.5**	**210 – 280**	**<3.0**	**≥15**	**25 – 37.4**	**>40**	**Yes**	**Yes**	NA	Other	No growth	In House
12	Terminal Sterilization	Radiophar macy	Pharmacist	**6.7–8.6**	**220 – 280**	**<3.0**	**≥15**	**25–37**	**>40**	**Yes**	**Yes**	<5.0	In House	No growth	In House
13	Sterile Preparation	Pharmacy /Radiopha rmacy	Pharmacist	**5 – 9**	**220 – 280**	**<3.0**	**≥15**	**25–37**	**>38**	NA	**Yes**	NA	In House	No growth	In House

**Table 2:** RF coil configurations reported for human HP [1-^13^C]pyruvate studies. TX = transmit coil, RX = receive coil. The commonly used “clamshell” TX coil is a Helmholz pair design. For ^1^H RF configurations, all used the Body coil for TX unless otherwise noted, and “repositioned” indicates the ^13^C coil was removed for ^1^H imaging. One representative reference is listed for each configuration. The RF coil configurations reported in the reviewed papers are shown in Supporting Table S1.

Anatomical Target	^13^C TX-transmit coil type	^13^C RX-receive coil type	^1^H coil setup	Representative Reference
Abdomen/Pelvis	clamshell coil	16-channel bilateral array	Body coil	(4)
Abdomen/Pelvis	clamshell coil	2× 4-channel paddle arrays	Body coil or 32-channel abdomen array, repositioned	(37)
Abdomen/Pelvis	clamshell coil	8-channel array	32-channel abdomen array, repositioned	(46), Fig 4A, B
Abdomen/Pelvis	semi-flexible quadrature coil	2× 4-channel arrays	Body coil	(62)
Brain	clamshell coil	2× 4-channel paddle arrays	8-channel head array, repositioned	(5)
Brain	birdcage coil	32-channel array	Body coil	(14), Fig 4C
Brain	quadrature coil	8-channel array	quadrature TX/RX head coil	(50)
Brain	dual-tuned 1H/^13^C quadrature coil	dual-tuned 1H/^13^C quadrature coil	dual-tuned 1H/^13^C quadrature coil	(55)
Brain	clamshell coil	8-channel receive array	Body coil	(57)
Breast	2-channel TX/RX	2-channel TX/RX and 6-channel RX-only	Body coil or 8-channel breast array, repositioned	(43)
Heart	clamshell coil	2× 4-channel paddle arrays	not reported	(2)
Heart	Helmholtz 2-loop coil-pair	Helmholtz 2-loop coil-pair	Body coil	(36)
Heart	semi-flexible quadrature coil	2× 4-channel arrays	Body coil	(36)
Heart	clamshell coil	16-channel receive array	Body coil or 8-channel cardiac array, repositioned	(52)
Prostate	clamshell coil	dual ^1^H/^13^C endorectal coil	dual ^1^H/^13^C endorectal coil	(1)

**Table 3:** Summary of the imaging systems, phantoms, and prescan procedures used at sites currently performing HP ^13^C-pyruvate human studies. These were obtained from a survey of all sites performing clinical trials with HP [1-^13^C]pyruvate. *Previously performed studies with a Siemens 3T Tim Trio. The imaging systems, phantoms, and prescan procedures reported in the reviewed papers are shown in Supporting Table S1.

Site	Primary Imaging System	^13^C Receive Channels	^13^C Peak Power [kW]	Phantom(s) - during study	Phantom(s) - before study	^13^C Frequency Calibration	Shimming	^13^C RF Transmit Power Calibration	^13^C RF Receive Gain Calibration
1	Philips 1.5T Achieva and 3T Ingenia	8	4	^13^C-urea, ^13^C-pyruvate, ^13^C-acetate, ^13^C-acetate, tubes	^13^C-urea, tube	Derived from ^1^H	Auto shimming	Power sweep on phantom before study	Not required
2	GE 3T Discovery MR750w	8	8	None	^13^C-urea doped with Gd, tube	Derived from ^1^H	Auto shimming	Bloch-Siegert	None
3	GE 3T Discovery MR750	8	8	^13^C-urea doped with Gd, sphere	^13^C-urea doped with Gd, sphere	Predetermined frequency	Auto shimming	Power sweep	Empirical
4	GE 3T Discovery MR750w	8	8	[1-^13^C]lactate doped with Gd None	^13^C-bicarbonate	Derived from ^1^H	PRESS box shim	Power sweep	Empirical
5	GE 3T Discovery MR750	32	8	^13^C-bicarbonate doped with Gd, sphere	ethylene glycol, head shape ethylene glycol, bottle dimethyl silicone, sphere & cuboid	Derived from ^1^H	Auto shimming High order shimming	Bloch-Siegert	Empirical
6	GE 3T Discovery MR750 and GE3TSigna PET/MR	32	8	^13^C-bicarbonate doped with Gd, sphere ^13^C-urea doped with Gd, bottle or Eppendorf tube	dimethyl silicone, various shapes ^13^C-bicarbonate doped with Gd, sphere ^13^C-urea doped with Gd, bottle or Eppendorftube	Derived from ^1^H	Auto shimming over PRESS box (breast, prostate) Auto shimming (abdomen) High order shimming (brain)	Bloch-Siegert	Empirical
7	GE 3T Discovery MR750w	8	8	^13^C-urea doped with Gd ethylene glycol	^13^C-urea doped with Gd, tube	Derived from ^1^H	Auto shimming followed by manual linear shimming	Power sweep	Empirical
8	GE 3T Signa Premier*	32	8	^13^C-urea doped with Gd, tube	ethylene glycol	Establishing protocol	Establishing protocol	Establishing protocol	Establishing Protocol
9	GE 3T Discovery MR750	32	11	^13^C-urea doped with Gd, tube	^13^C-urea doped with Gd, sphere ethylene glycol, head shape dimethyl silicone, sphere & cuboid	Derived from ^1^H	Auto shimming Specialized shimming	Power sweep	Empirical
10	Siemens 3T Biograph mMR	12		^13^C-urea doped with Gd, tube	^13^C-urea doped with Gd, tube ethylene glycol	Based on ^13^C phantom	Auto shimming	Power sweep	Automatic
11	GE 3T Signa Premier	32	8	^13^C-urea doped with Gd, tube	dimethyl silicone, cylinders	Based on ^13^C phantom	Auto shimming	RF pulse sweep	Maximum values
12	GE 3T Discovery MR750	8	8	^13^C-urea in D2O doped with Gd	dimethyl silicone, various shapes ethylene glycol, various shapes	Derived from ^1^H	Auto shimming	Power sweep Bloch-Siegert	Maximum values
13	GE 3T Discovery MR750w	8	8	^13^C-urea doped with Gd	ethylene glycol, sphere	Based on ^13^C phantom	Auto shimming	Bloch-Siegert, checked with power sweep	Maximum values

## References

[1] Nelson SJ, Kurhanewicz J, Vigneron DB, Metabolic Imaging of Patients with Prostate Cancer Using Hyperpolarized [1–13C]Pyruvate. Sci Transl Med. 2013;5(198):198ra108. doi:10.1126/scitranslmed.3006070 PMC4201045 23946197

[2] Cunningham CH, Lau JYC, Chen AP, Hyperpolarized 13C Metabolic MRI of the Human HeartNovelty and Significance: Initial Experience. Circ Res. 2016;119(11):1177–1182. doi:10.1161/CIRCRESAHA.116.309769 27635086 PMC5102279

[3] Aggarwal R, Vigneron DB, Kurhanewicz J. Hyperpolarized 1-[13C]-Pyruvate Magnetic Resonance Imaging Detects an Early Metabolic Response to Androgen Ablation Therapy in Prostate Cancer. European Urology. 2017;72(6):1028–1029. doi:10.1016/j.eururo.2017.07.022 28765011 PMC5723206

[4] Gordon JW, Hansen RB, Shin PJ, Feng Y, Vigneron DB, Larson PEZ. 3D hyperpolarized C-13 EPI with calibrationless parallel imaging. J Magn Reson. 2018;289:92–99. doi:10.1016/j.jmr.2018.02.011 29476930 PMC5856653

[5] Miloushev VZ, Granlund KL, Boltyanskiy R, Metabolic Imaging of the Human Brain with Hyperpolarized 13C Pyruvate Demonstrates 13C Lactate Production in Brain Tumor Patients. Cancer Res. 2018;78(14):3755–3760. doi:10.1158/0008-5472.CAN-18-0221 29769199 PMC6050093

[6] Park I, Larson PEZ, Gordon JW, Development of methods and feasibility of using hyperpolarized carbon-13 imaging data for evaluating brain metabolism in patient studies. Magnetic Resonance in Medicine. 2018;80(3):864–873. doi:10.1002/mrm.27077 29322616 PMC5980662

[7] Chen HY, Larson PEZ, Gordon JW, Technique development of 3D dynamic CS-EPSI for hyperpolarized 13 C pyruvate MR molecular imaging of human prostate cancer. Magn Reson Med. 2018;80(5):2062–2072. doi:10.1002/mrm.27179 29575178 PMC6107425

[8] Larson PEZ, Chen HY, Gordon JW, Investigation of analysis methods for hyperpolarized 13C-pyruvate metabolic MRI in prostate cancer patients. NMR Biomed. 2018;31(11):e3997. doi:10.1002/nbm.3997 30230646 PMC6392436

[9] Maidens J, Gordon JW, Chen HY, Spatio-Temporally Constrained Reconstruction for Hyperpolarized Carbon-13 MRI Using Kinetic Models. IEEE Trans Med Imaging. 2018;37(12):2603–2612. doi:10.1109/TMI.2018.2844246 29994332 PMC6279499

[10] Gordon JW, Chen HY, Autry A, Translation of Carbon-13 EPI for hyperpolarized MR molecular imaging of prostate and brain cancer patients. Magn Reson Med. 2019;81(4):2702–2709. doi:10.1002/mrm.27549 30375043 PMC6372313

[11] Zhu Z, Zhu X, Ohliger MA, Coil combination methods for multi-channel hyperpolarized 13C imaging data from human studies. J Magn Reson. 2019;301:73–79. doi:10.1016/j.jmr.2019.01.015 30851668 PMC7170546

[12] Grist JT, McLean MA, Riemer F, Quantifying normal human brain metabolism using hyperpolarized [1–13C]pyruvate and magnetic resonance imaging. Neuroimage. 2019;189:171–179. doi:10.1016/j.neuroimage.2019.01.027 30639333 PMC6435102

[13] Tran M, Latifoltojar A, Neves JB, First-in-human in vivo non-invasive assessment of intra-tumoral metabolic heterogeneity in renal cell carcinoma. BJR Case Rep. 2019;5(3):20190003. doi:10.1259/bjrcr.20190003 31428445 PMC6699984

[14] Autry AW, Gordon JW, Carvajal L, Comparison between 8- and 32-channel phased-array receive coils for in vivo hyperpolarized 13 C imaging of the human brain. Magn Reson Med. 2019;82(2):833–841. doi:10.1002/mrm.27743 30927300 PMC6612511

[15] Abeyakoon O, Latifoltojar A, Gong F, Hyperpolarised 13 C MRI: a new horizon for non-invasive diagnosis of aggressive breast cancer. BJR Case Rep. 2019;5(3):20190026. doi:10.1259/bjrcr.20190026 31555479 PMC6750630

[16] Autry AW, Gordon JW, Chen HY, Characterization of serial hyperpolarized 13C metabolic imaging in patients with glioma. Neuroimage Clin. 2020;27:102323. doi:10.1016/j.nicl.2020.102323 32623139 PMC7334458

[17] Lee CY, Soliman H, Geraghty BJ, Lactate topography of the human brain using hyperpolarized 13C-MRI. Neuroimage. 2020;204:116202. doi:10.1016/j.neuroimage.2019.116202 31557546

[18] Granlund KL, Tee SS, Vargas HA, Hyperpolarized MRI of Human Prostate Cancer Reveals Increased Lactate with Tumor Grade Driven by Monocarboxylate Transporter 1. Cell Metab. 2020;31(1):105–114.e3. doi:10.1016/j.cmet.2019.08.024 31564440 PMC6949382

[19] Gallagher FA, Woitek R, McLean MA, Imaging breast cancer using hyperpolarized carbon-13 MRI. Proc Natl Acad Sci U S A. 2020;117(4):2092–2098. doi:10.1073/pnas.1913841117 31964840 PMC6995024

[20] Mammoli D, Gordon J, Autry A, Kinetic Modeling of Hyperpolarized Carbon-13 Pyruvate Metabolism in the Human Brain. IEEE Trans Med Imaging. 2020;39(2):320–327. doi:10.1109/TMI.2019.2926437 31283497 PMC6939147

[21] Stødkilde-Jørgensen H, Laustsen C, Hansen ESS, Pilot Study Experiences With Hyperpolarized [1–13 C]pyruvate MRI in Pancreatic Cancer Patients. J Magn Reson Imaging. 2020;51(3):961–963. doi:10.1002/jmri.26888 31368215

[22] Rider OJ, Apps A, Miller JJJJ, Noninvasive In Vivo Assessment of Cardiac Metabolism in the Healthy and Diabetic Human Heart Using Hyperpolarized 13C MRI. Circ Res. 2020;126(6):725–736. doi:10.1161/CIRCRESAHA.119.316260 32078413 PMC7077975

[23] Chen HY, Aggarwal R, Bok RA, Hyperpolarized 13C-pyruvate MRI detects real-time metabolic flux in prostate cancer metastases to bone and liver: a clinical feasibility study. Prostate Cancer Prostatic Dis. 2020;23(2):269–276. doi:10.1038/s41391-019-0180-z 31685983 PMC7196510

[24] Woitek R, McLean MA, Gill AB, Hyperpolarized 13C MRI of Tumor Metabolism Demonstrates Early Metabolic Response to Neoadjuvant Chemotherapy in Breast Cancer. Radiol Imaging Cancer. 2020;2(4):e200017. doi:10.1148/rycan.2020200017 32803167 PMC7398116

[25] Tang S, Bok R, Qin H, A metabolite-specific 3D stack-of-spiral bSSFP sequence for improved lactate imaging in hyperpolarized [1–13 C]pyruvate studies on a 3T clinical scanner. Magn Reson Med. 2020;84(3):1113–1125. doi:10.1002/mrm.28204 32086845 PMC7927572

[26] Walker CM, Gordon JW, Xu Z, Slice profile effects on quantitative analysis of hyperpolarized pyruvate. NMR Biomed. 2020;33(10):e4373. doi:10.1002/nbm.4373 32743881 PMC7484340

[27] Chen HY, Autry AW, Brender JR, Tensor image enhancement and optimal multichannel receiver combination analyses for human hyperpolarized 13 C MRSI. Magn Reson Med. 2020;84(6):3351–3365. doi:10.1002/mrm.28328 32501614 PMC7718428

[28] Gordon JW, Autry AW, Tang S, A variable resolution approach for improved acquisition of hyperpolarized 13 C metabolic MRI. Magn Reson Med. 2020;84(6):2943–2952. doi:10.1002/mrm.28421 32697867 PMC7719570

[29] Ma J, Park JM. Super-Resolution Hyperpolarized 13C Imaging of Human Brain Using Patch-Based Algorithm. Tomography. 2020;6(4):343–355. doi:10.18383/j.tom.2020.00037 33364424 PMC7744189

[30] Park JM, Reed GD, Liticker J, Effect of Doxorubicin on Myocardial Bicarbonate Production From Pyruvate Dehydrogenase in Women With Breast Cancer. Circ Res. 2020;127(12):1568–1570. doi:10.1161/CIRCRESAHA.120.317970 33054563 PMC7874930

[31] Hackett EP, Pinho MC, Harrison CE, Imaging Acute Metabolic Changes in Patients with Mild Traumatic Brain Injury Using Hyperpolarized [1–13C]Pyruvate. iScience. 2020;23(12):101885. doi:10.1016/j.isci.2020.101885 33344923 PMC7736977

[32] Chen J, Patel TR, Pinho MC, Preoperative imaging of glioblastoma patients using hyperpolarized 13C pyruvate: Potential role in clinical decision making. Neurooncol Adv. 2021;3(1):vdab092. doi:10.1093/noajnl/vdab092 34355174 PMC8331053

[33] Autry AW, Park I, Kline C, Pilot Study of Hyperpolarized 13C Metabolic Imaging in Pediatric Patients with Diffuse Intrinsic Pontine Glioma and Other CNS Cancers. AJNR Am J Neuroradiol. 2021;42(1):178–184. doi:10.3174/ajnr.A6937 33272950 PMC7814790

[34] Lee CY, Soliman H, Bragagnolo ND, Predicting response to radiotherapy of intracranial metastases with hyperpolarized 13C MRI. J Neurooncol. 2021;152(3):551–557. doi:10.1007/s11060-021-03725-7 33740165 PMC8084843

[35] Apps A, Lau JYC, Miller JJJJ, Proof-of-Principle Demonstration of Direct Metabolic Imaging Following Myocardial Infarction Using Hyperpolarized 13C CMR. JACC Cardiovasc Imaging. 2021;14(6):1285–1288. doi:10.1016/j.jcmg.2020.12.023 33582059 PMC8184499

[36] Reed GD, Ma J, Park JM, Characterization and compensation of f0 inhomogeneity artifact in spiral hyperpolarized 13 C imaging of the human heart. Magn Reson Med. 2021;86(1):157–166. doi:10.1002/mrm.28691 33547689 PMC8049085

[37] Tang S, Meng MV, Slater JB, Metabolic imaging with hyperpolarized 13 C pyruvate magnetic resonance imaging in patients with renal tumors-Initial experience. Cancer. 2021;127(15):2693–2704. doi:10.1002/cncr.33554 33844280 PMC8565774

[38] Ma J, Chen J, Reed GD, Cardiac T2∗ measurement of hyperpolarized 13 C metabolites using metabolite-selective multi-echo spiral imaging. Magn Reson Med. 2021;86(3):1494–1504. doi:10.1002/mrm.28796 33821504 PMC8212421

[39] Park JM, Harrison CE, Ma J, Hyperpolarized 13C MR Spectroscopy Depicts in Vivo Effect of Exercise on Pyruvate Metabolism in Human Skeletal Muscle. Radiology. 2021;300(3):626–632. doi:10.1148/radiol.2021204500 34156298 PMC8409104

[40] Bøgh N, Olin RB, Hansen ES, Metabolic MRI with hyperpolarized [1–13C]pyruvate separates benign oligemia from infarcting penumbra in porcine stroke. J Cereb Blood Flow Metab. 2021;41(11):2916–2927. doi:10.1177/0271678X211018317 34013807 PMC8756460

[41] Kim Y, Chen HY, Autry AW, Denoising of hyperpolarized 13 C MR images of the human brain using patch-based higher-order singular value decomposition. Magn Reson Med. 2021;86(5):2497–2511. doi:10.1002/mrm.28887 34173268 PMC8530853

[42] Lee PM, Chen HY, Gordon JW, Specialized computational methods for denoising, B1 correction, and kinetic modeling in hyperpolarized 13 C MR EPSI studies of liver tumors. Magn Reson Med. 2021;86(5):2402–2411. doi:10.1002/mrm.28901 34216051 PMC8565779

[43] Woitek R, McLean MA, Ursprung S, Hyperpolarized Carbon-13 MRI for Early Response Assessment of Neoadjuvant Chemotherapy in Breast Cancer Patients. Cancer Res. 2021;81(23):6004–6017. doi:10.1158/0008-5472.CAN-21-1499 34625424 PMC7612070

[44] Vaziri S, Autry AW, Lafontaine M, Assessment of higher-order singular value decomposition denoising methods on dynamic hyperpolarized [1–13C]pyruvate MRI data from patients with glioma. Neuroimage Clin. 2022;36:103155. doi:10.1016/j.nicl.2022.103155 36007439 PMC9421383

[45] Ma J, Malloy CR, Pena S, Dual-phase imaging of cardiac metabolism using hyperpolarized pyruvate. Magn Reson Med. 2022;87(1):302–311. doi:10.1002/mrm.29042 34617626 PMC8616832

[46] Ursprung S, Woitek R, McLean MA, Hyperpolarized 13C-Pyruvate Metabolism as a Surrogate for Tumor Grade and Poor Outcome in Renal Cell Carcinoma-A Proof of Principle Study. Cancers (Basel). 2022;14(2):335. doi:10.3390/cancers14020335 35053497 PMC8773685

[47] Sushentsev N, McLean MA, Warren AY, Hyperpolarised 13C-MRI identifies the emergence of a glycolytic cell population within intermediate-risk human prostate cancer. Nat Commun. 2022;13(1):466. doi:10.1038/s41467-022-28069-2 35075123 PMC8786834

[48] de Kouchkovsky I, Chen HY, Ohliger MA, Hyperpolarized 1-[13C]-Pyruvate Magnetic Resonance Imaging Detects an Early Metabolic Response to Immune Checkpoint Inhibitor Therapy in Prostate Cancer. Eur Urol. 2022;81(2):219–221. doi:10.1016/j.eururo.2021.10.015 34758912 PMC9275093

[49] Ehrhardt MJ, Gallagher FA, McLean MA, Schönlieb CB. Enhancing the spatial resolution of hyperpolarized carbon-13 MRI of human brain metabolism using structure guidance. Magn Reson Med. 2022;87(3):1301–1312. doi:10.1002/mrm.29045 34687088

[50] Ma J, Pinho MC, Harrison CE, Dynamic 13 C MR spectroscopy as an alternative to imaging for assessing cerebral metabolism using hyperpolarized pyruvate in humans. Magn Reson Med. 2022;87(3):1136–1149. doi:10.1002/mrm.29049 34687086 PMC8776582

[51] Bøgh N, Gordon JW, Hansen ESS, Initial Experience on Hyperpolarized [1–13C]Pyruvate MRI Multicenter Reproducibility-Are Multicenter Trials Feasible? Tomography. 2022;8(2):585–595. doi:10.3390/tomography8020048 35314625 PMC8938827

[52] Joergensen SH, Hansen ESS, Bøgh N, Detection of increased pyruvate dehydrogenase flux in the human heart during adenosine stress test using hyperpolarized [1–13C]pyruvate cardiovascular magnetic resonance imaging. J Cardiovasc Magn Reson. 2022;24(1):34. doi:10.1186/s12968-022-00860-6 35658896 PMC9169396

[53] Bøgh N, Laustsen C, Hansen ESS, Tankisi H, Bertelsen LB, Blicher JU. Imaging Neurodegenerative Metabolism in Amyotrophic Lateral Sclerosis with Hyperpolarized [1–13C]pyruvate MRI. Tomography. 2022;8(3):1570–1577. doi:10.3390/tomography8030129 35736877 PMC9231312

[54] Zaccagna F, McLean MA, Grist JT, Imaging Glioblastoma Metabolism by Using Hyperpolarized [1–13C]Pyruvate Demonstrates Heterogeneity in Lactate Labeling: A Proof of Principle Study. Radiol Imaging Cancer. 2022;4(4):e210076. doi:10.1148/rycan.210076 35838532 PMC9360994

[55] Kaggie JD, Khan AS, Matys T, Deuterium metabolic imaging and hyperpolarized 13C-MRI of the normal human brain at clinical field strength reveals differential cerebral metabolism. Neuroimage. 2022;257:119284. doi:10.1016/j.neuroimage.2022.119284 35533826

[56] Liu X, Tang S, Mu C, Development of specialized magnetic resonance acquisition techniques for human hyperpolarized [13 C,15 N2 ]urea + [1–13 C]pyruvate simultaneous perfusion and metabolic imaging. Magn Reson Med. 2022;88(3):1039–1054. doi:10.1002/mrm.29266 35526263 PMC9810116

[57] Sanchez-Heredia JD, Olin RB, Grist JT, RF coil design for accurate parallel imaging on 13 C MRSI using 23 Na sensitivity profiles. Magn Reson Med. 2022;88(3):1391–1405. doi:10.1002/mrm.29259 35635156 PMC9328386

[58] Sushentsev N, McLean MA, Warren AY, The potential of hyperpolarised 13C-MRI to target glycolytic tumour core in prostate cancer. Eur Radiol. 2022;32(10):7155–7162. doi:10.1007/s00330-022-08929-7 35731287 PMC9474577

[59] Chowdhury R, Mueller CA, Smith L, Quantification of Prostate Cancer Metabolism Using 3D Multiecho bSSFP and Hyperpolarized [1–13 C] Pyruvate: Metabolism Differs Between Tumors of the Same Gleason Grade. J Magn Reson Imaging. Published online October 31, 2022. doi:10.1002/jmri.28467 PMC10946772 36315000

[60] Hu JY, Kim Y, Autry AW, Kinetic analysis of multi-resolution hyperpolarized 13 C human brain MRI to study cerebral metabolism. Magn Reson Med. 2022;88(5):2190–2197. doi:10.1002/mrm.29354 35754148 PMC9420752

[61] Chen HY, Bok RA, Cooperberg MR, Improving multiparametric MR-transrectal ultrasound guided fusion prostate biopsies with hyperpolarized 13 C pyruvate metabolic imaging: A technical development study. Magn Reson Med. 2022;88(6):2609–2620. doi:10.1002/mrm.29399 35975978 PMC9794017

[62] Lee PM, Chen HY, Gordon JW, Whole-Abdomen Metabolic Imaging of Healthy Volunteers Using Hyperpolarized [1–13 C]pyruvate MRI. J Magn Reson Imaging. 2022;56(6):1792–1806. doi:10.1002/jmri.28196 35420227 PMC9562149

[63] Miloushev VZ, Boltyanskiy R, Granlund KL, Keshari KR. Improved total sensitivity estimation for multiple receive coils in MRI using ratios of first-order statistics. MAGMA. 2022;35(6):895–901. doi:10.1007/s10334-022-01028-0 35876917 PMC9613615

[64] Chung BT, Chen HY, Gordon J, First hyperpolarized [2–13C]pyruvate MR studies of human brain metabolism. Journal of Magnetic Resonance. 2019;309:106617. doi:10.1016/j.jmr.2019.106617 31648132 PMC6880930

[65] Vanderheyden JL. Summary of Data-Non-clinical Hyperpolarized Pyruvate (13C) Injection. https://imaging.cancer.gov/programs_resources/cancer-tracer-synthesis-resources/docs/c13-pyruvate-non-clinical-summary.pdf

[66] Jacobs PM. Summary of Data: Clinical Hyperpolarized Pyruvate (13C) Injection. https://imaging.cancer.gov/programs_resources/cancer-tracer-synthesis-resources/docs/c13-pyruvate-clinical-data-summary.pdf

[67] Ardenkjaer-Larsen JH, Leach AM, Clarke N, Urbahn J, Anderson D, Skloss TW. Dynamic nuclear polarization polarizer for sterile use intent. NMR in Biomedicine. 2011;24(8):927–932. doi:10.1002/nbm.1682 21416540

[68] Hyperpolarized Pyruvate (13C) Injection: Investigator’s Brochure. https://imaging.cancer.gov/programs_resources/cancer-tracer-synthesis-resources/docs/c13_pyruvate_IB_PDF.pdf

[69] USP General Chapter 823 - Positron Emission Tomography Drugs for Compounding, Investigational, and Research Uses. Accessed February 13, 2023. https://www.uspnf.com/sites/default/files/usp_pdf/EN/USPNF/key-issues/usp35-nf30_general_chapter_823.pdf

[70] USP General Chapter 797 - Pharmaceutical Compounding - Sterile Preparations. Accessed February 13, 2023. https://www.usp.org/compounding/general-chapter-797 15181114

[71] Fedotov A. FDA and EU GMP Annex 1 Differences in Cleanroom Specifications. Is it time to eliminate them? Regulatory Focus (Regulatory Affairs Professionals Society). Published online July 2019. https://www.raps.org/news-and-articles/news-articles/2019/7/fda-and-eu-gmp-annex-1-differences-in-cleanroom-sp

[72] Ferrone M. SAMPLE CMC SECTION FOR HYPERPOLARIZED PYRUVATE (13C) INJECTION. Accessed February 13, 2023. https://imaging.cancer.gov/programs_resources/cancer-tracer-synthesis-resources/docs/c13_pyruvate_sample_CMC_from_UCSF.pdf

[73] Traechtler J, Fuetterer M, Albannay MM, Hoh T, Kozerke S. Considerations for hyperpolarized 13C MR at reduced field: Comparing 1.5T versus 3T. Magnetic Resonance in Medicine. 2023;89(5):1945–1960. doi:10.1002/mrm.29579 36598063

[74] McLean MA, Hinks RS, Kaggie JD, Characterization and correction of center-frequency effects in X-nuclear eddy current compensations on a clinical MR system. Magnetic Resonance in Medicine. 2021;85(5):2370–2376. doi:10.1002/mrm.28607 33274790 PMC7898706

[75] Sanchez-Heredia JD, Szocska Hansen ES, Laustsen C, Zhurbenko V, Ardenkjær-Larsen JH. Low-Noise Active Decoupling Circuit and its Application to 13C Cryogenic RF Coils at 3 T. Tomography. 2017;3(1):60–66. doi:10.18383/j.tom.2016.00280 30042972 PMC6024423

[76] Harlan CJ, Xu Z, Walker CM, Michel KA, Reed GD, Bankson JA. The Effect of Transmit B1 Inhomogeneity on Hyperpolarized [1–13C]-Pyruvate Metabolic MR Imaging Biomarkers. Medical Physics. n/a(n/a). doi:10.1002/mp.15107 PMC8486175 34287945

[77] Price R, Allison J, Clarke G, Magnetic Resonance Imaging Quality Control Manual. Published online 2015. https://www.acr.org/-/media/ACR/Files/Clinical-Resources/QC-Manuals/MR_QCManual.pdf

[78] Sánchez-Heredia JD, Olin RB, McLean MA, Multi-site benchmarking of clinical 13C RF coils at 3T. J Magn Reson. 2020;318:106798. doi:10.1016/j.jmr.2020.106798 32755748 PMC7501735

[79] Walker CM, Lee J, Ramirez MS, Schellingerhout D, Millward S, Bankson JA. A catalyzing phantom for reproducible dynamic conversion of hyperpolarized [1-(13)c]-pyruvate. PLoS One. 2013;8(8):e71274. doi:10.1371/journal.pone.0071274 23977006 PMC3744565

[80] Chowdhury R, Papoutsaki MV, Müller CA, A reproducible dynamic phantom for sequence testing in hyperpolarised 13C-magnetic resonance. Br J Radiol. 2022;95(1134):20210770. doi:10.1259/bjr.20210770 35230136 PMC10996405

[81] Daniels CJ, McLean MA, Schulte RF, A comparison of quantitative methods for clinical imaging with hyperpolarized 13C-pyruvate. NMR Biomed. 2016;29(4):387–399. doi:10.1002/nbm.3468 27414749 PMC4833181

[82] Grist JT, Hansen ESS, Sánchez-Heredia JD, Creating a clinical platform for carbon-13 studies using the sodium-23 and proton resonances. Magnetic Resonance in Medicine. 2020;84(4):1817–1827. doi:10.1002/mrm.28238 32167199 PMC8638662

[83] Tang S, Milshteyn E, Reed G, A regional bolus tracking and real-time B1 calibration method for hyperpolarized 13 C MRI. Magn Reson Med. 2019;81(2):839–851. doi:10.1002/mrm.27391 30277268 PMC6289616

[84] Schulte RF, Sacolick L, Deppe MH, Transmit gain calibration for nonproton MR using the Bloch-Siegert shift. NMR Biomed. 2011;24(9):1068–1072. doi:10.1002/nbm.1657 21387441

[85] Wiesinger F, Weidl E, Menzel MI, IDEAL spiral CSI for dynamic metabolic MR imaging of hyperpolarized [1–13C]pyruvate. Magn Reson Med. 2012;68(1):8–16. doi:10.1002/mrm.23212 22127962

[86] Cunningham CH, Chen AP, Lustig M, Pulse sequence for dynamic volumetric imaging of hyperpolarized metabolic products. J Magn Reson. 2008;193(1):139–146. 18424203 10.1016/j.jmr.2008.03.012 PMC3051833

[87] Hansen RB, Sánchez-Heredia JD, Bøgh N, Coil profile estimation strategies for parallel imaging with hyperpolarized 13C MRI. Magnetic Resonance in Medicine. 2019;82(6):2104–2117. doi:10.1002/mrm.27892 31297868

[88] Brittain JH, Olcott EW, Szuba A, Three-dimensional flow-independent peripheral angiography. Magn Reson Med. 1997;38(3):343–354. doi:10.1002/mrm.1910380302 9339435

[89] Wattjes MP, Ciccarelli O, Reich DS, 2021 MAGNIMS-CMSC-NAIMS consensus recommendations on the use of MRI in patients with multiple sclerosis. Lancet Neurol. 2021;20(8):653–670. doi:10.1016/S1474-4422(21)00095-8 34139157

[90] Boellaard R. Standards for PET image acquisition and quantitative data analysis. J Nucl Med. 2009;50 Suppl 1:11S–20S. doi:10.2967/jnumed.108.057182 19380405

[91] Lu J, Wang Z, Bier E, Leewiwatwong S, Mummy D, Driehuys B. Bias field correction in hyperpolarized 129 Xe ventilation MRI using templates derived by RF-depolarization mapping. Magn Reson Med. 2022;88(2):802–816. doi:10.1002/mrm.29254 35506520 PMC9248357

[92] Xu T, Mayer D, Gu M, Quantification of in vivo metabolic kinetics of hyperpolarized pyruvate in rat kidneys using dynamic 13C MRSI. NMR in Biomedicine. 2011;24(8):997–1005. doi:10.1002/nbm.1719 21538639 PMC3169748

[93] Hill DK, Orton MR, Mariotti E, Model free approach to kinetic analysis of real-time hyperpolarized 13C magnetic resonance spectroscopy data. PLoS One. 2013;8(9):e71996. doi:10.1371/journal.pone.0071996 24023724 PMC3762840

[94] Day SE, Kettunen MI, Gallagher FA, Detecting tumor response to treatment using hyperpolarized 13C magnetic resonance imaging and spectroscopy. Nat Med. 2007;13(11):1382–1387. doi:10.1038/nm1650 17965722

[95] Kazan SM, Reynolds S, Kennerley A, Kinetic modeling of hyperpolarized (13)C pyruvate metabolism in tumors using a measured arterial input function. Magn Reson Med. 2013;70(4):943–953. doi:10.1002/mrm.24546 23169010

[96] Khegai O, Schulte RF, Janich MA, Apparent rate constant mapping using hyperpolarized [1-(13)C]pyruvate. NMR Biomed. 2014;27(10):1256–1265. doi:10.1002/nbm.3174 25156807

[97] Zierhut ML, Yen YF, Chen AP, Kinetic modeling of hyperpolarized 13C1-pyruvate metabolism in normal rats and TRAMP mice. J Magn Reson. 2010;202(1):85–92. doi:10.1016/j.jmr.2009.10.003 19884027 PMC2833325

[98] Bankson JA, Walker CM, Ramirez MS, Kinetic Modeling and Constrained Reconstruction of Hyperpolarized [1–13C]-Pyruvate Offers Improved Metabolic Imaging of Tumors. Cancer Res. 2015;75(22):4708–4717. doi:10.1158/0008-5472.CAN-15-0171 26420214 PMC4651725

[99] Menichetti L, Frijia F, Flori A, Assessment of real-time myocardial uptake and enzymatic conversion of hyperpolarized [1-^13^C]pyruvate in pigs using slice selective magnetic resonance spectroscopy. Contrast Media Mol Imaging. 2012;7(1):85–94. doi:10.1002/cmmi.480 22344884

[100] Hyperpolarized-MRI-Toolbox. doi:10.5281/zenodo.1198915

[101] Miller JJ, Grist JT, Serres S, 13C Pyruvate Transport Across the Blood-Brain Barrier in Preclinical Hyperpolarised MRI. Sci Rep. 2018;8(1):15082. doi:10.1038/s41598-018-33363-5 30305655 PMC6180068

[102] Chaumeil MM, Radoul M, Najac C, Hyperpolarized (13)C MR imaging detects no lactate production in mutant IDH1 gliomas: Implications for diagnosis and response monitoring. Neuroimage Clin. 2016;12:180–189. doi:10.1016/j.nicl.2016.06.018 27437179 PMC4939422

[103] Schroeder MA, Lau AZ, Chen AP, Hyperpolarized 13C magnetic resonance reveals early- and late-onset changes to in vivo pyruvate metabolism in the failing heart. European Journal of Heart Failure. 2013;15(2):130–140. doi:10.1093/eurjhf/hfs192 23258802 PMC3547367

[104] Mariager CØ, Lindhardt J, Nielsen PM, Schulte RF, Ringgaard S, Laustsen C. Fractional Perfusion: A Simple Semi-Parametric Measure for Hyperpolarized 13C MR. IEEE Transactions on Radiation and Plasma Medical Sciences. 2019;3(4):523–527. doi:10.1109/TRPMS.2019.2905724

[105] Mikkelsen EFR, Mariager CØ, Nørlinger T, Hyperpolarized [1–13C]-acetate Renal Metabolic Clearance Rate Mapping. Sci Rep. 2017;7(1):16002. doi:10.1038/s41598-017-15929-x 29167446 PMC5700138

[106] Borland D, Taylor MR. Rainbow color map (still) considered harmful. IEEE Comput Graph Appl. 2007;27(2):14–17. doi:10.1109/mcg.2007.323435 17388198

[107] Nuñez JR, Anderton CR, Renslow RS. Optimizing colormaps with consideration for color vision deficiency to enable accurate interpretation of scientific data. PLoS One. 2018;13(7):e0199239. doi:10.1371/journal.pone.0199239 30067751 PMC6070163

[108] Ferrari A, Peters J, Anikeeva M, Performance and reproducibility of 13C and 15N hyperpolarization using a cryogen-free DNP polarizer. Sci Rep. 2022;12(1):11694. doi:10.1038/s41598-022-15380-7 35803961 PMC9270333

