# Supplementary material for: Current Methods for Hyperpolarized [1-13C]pyruvate MRI Human Studies
Source: ArXiv. 2023 Sep 7:arXiv:2309.04040v1. Preprint. [Version 1] (PMC10508833)
Supplement: Supplementary file 1 [file NIHPP2309.04040V1-supplement-1.pdf]

Supporting Tables

Supporting Table S1: MRI system setup and calibration methods reported in HP ^{13}C-pyruvate human study papers surveyed. The entries are blank when the methods were not reported in the manuscript or supplementary/supporting materials.

Supporting Table S2: Acquisition methods reported in HP ^{13}C-pyruvate human study papers surveyed. Note that some papers reported multiple acquisition methods. The entries are blank when the methods were not reported in the manuscript or supplementary/supporting materials. If some values were not reported, they are denoted by a "?". In some cases, the methods were ambiguous and have been inferred from the cited paper or its references, which are *italicized* and followed by "?". "~" indicates a range of parameter values were reported. N/A = Not applicable. For the Start Time, some studies reported a single value, but it was not clear whether this was relative to the start of injection, end of injection, or other time, and these are shown as a single value and are *italicized*.

Supporting Table S3: Metrics reported in HP ^{13}C-pyruvate human study papers surveyed.

Supporting Information for "Current Methods for Hyperpolarized [1-13C]pyruvate MRI Human Studies"

Supporting Table S1: MRI system setup and calibration methods reported in HP ^{13}C-pyruvate human study papers surveyed. The entries are blank when the methods were not reported in the manuscript or supplementary/supporting materials.
Abbreviations: TX = transmit coil; RX = receive coil; TG = transmit gain; CF = center frequency
Coil Vendors: GE = GE Healthcare, Waukesha, WI, USA; RAPID Biomedical = RAPID Biomedical GmbH, Rimpar, Germany; Clinical MR Solutions = Clinical MR Solutions, Brookfield, WI, USA; Invivo = Invivo Inc.; PulseTeq = PulseTeq Limited, Chobham, Surrey, UK

Ref	Imaging System	RF coils 13C	RF coils 1H	Phantoms	Pre-scan
1	GE 3T Discovery MR750	TX 13C clamshell<br>RX 1H/13C endorectal coil (GE)	1H body coil, 1H/13C endorectal coil (GE)	8M 13C urea	signal calibration using the 8M 13C urea phantom
2	GE 3T Discovery MR750	TX 13C volume transmit coil system (GE)<br>RX Two 4-channel 13C surface coils arrays (GE)		spherical phantom containing an ≈8 mol/L solution of 13C-urea, which was fixed on top of the anterior receiver coil housing	prescan calibration of the 13C receive frequency and transmit power was performed using the signal from a 1.5-cm diameter spherical phantom
3	GE 3T Discovery MR750				
4	GE 3T Discovery MR750	TX 13C clamshell (GE)<br>RX 16-channel bilateral phased array (RAPID)			
5	GE 3T Discovery MR750w	TX 13C clamshell (GE)<br>RX Two 4-channel 13C surface coils arrays (GE)	8-channel 1H transmit/receive head coil		
6	GE 3T Discovery MR750	TX 13C clamshell (GE)<br>RX Two 4-channel 13C surface coils arrays (GE)		unenriched ethylene glycol + sealed standard that is housed within one of the eight-channel phased array elements and contains 1 mL of 8 M of 13C-urea.	Frequency calibration and B1+ map with phantom
7	GE 3T Discovery MR750	TX 13C clamshell<br>RX 1H/13C endorectal coil (GE)	4-channel pelvic phased coil array + 1H-tuned endorectal coil element	13C-urea phantom positioned on the receive coil (8 M, 600 μL) and 2 ethylene glycol phantoms (natural abundance, 13C concentration = 0.17 M)	13C-urea reference used for 13C RF calibration and center frequency, 1H MRSI shimming
8	GE 3T Discovery MR750				
9	GE 3T Discovery MR750				
10	GE 3T Discovery MR750	TX 13C clamshell (GE)<br>RX 1H/13C endorectal coil (GE)<br>or a 32-channel head array coil with integrated birdcage transmit coil	1H/13C endorectal coil	8 M 13C urea phantom	center frequency was calibrated using 8 M 13C-urea standard
11	GE 3T Discovery MR750	TX 13C clamshell (GE)<br>RX 16-channel bilateral phased array (RAPID)<br>or custom-built 32-channel 13C coil			

12	GE 3T Discovery MR750	dual-tuned 1H/13C quadrature head coil (RAPID)	dual-tuned 1H/13C quadrature head coil (RAPID)	8 M 13C urea attached to the ear defenders worn by the subject	13C transmit gain (TG) and center frequency (f0) were set using a13C enriched urea phantom
13	Siemens 3T Biograph mMR	TX 13C clamshell<br>RX two (anterior and posterior) 7-channel 1H/13C receive phased array coils (RAPID)	TX 1H body coil<br>RX two (anterior and posterior)<br>7-channel 1 h/13C receive phased array coils (RAPID)		
14	GE 3T Discovery MR750	TX 13C clam-shell coil (GE)<br>RX Two 4-channel 13C surface coils arrays (GE)<br>or a 32-channel head array coil with integrated birdcage transmit coil	body coil	A head-shaped phantom containing unenriched ethylene glycol doped with 17 g/L (0.29 M) NaCl	center frequency and calibrate power (TG) with a non-slice selective 90° or 180° RF pulse, B1+ map of head phantom
15	Siemens 3T Biograph mMR	TX 13C clamshell RX two (anterior and posterior) 7-channel 1 h/13C receive phased array coils (RAPID)	TX 1H body coil<br>RX two (anterior and posterior)<br>7-channel 1 h/13C receive phased array coils (RAPID)	reference phantom containing 1 ml of 8M 13C-urea	The 13C receiver bandwidth was centered using a reference phantom
16	GE 3T Discovery MR750	8-channel 13C RX / clamshell TX<br>32-channel 13C RX / Volume TX<br>8/24-channel 1H/13C RX / Volume TX	body coil or dual-tuned 13C/1H hardware	A head-shaped phantom containing unenriched ethylene glycol doped with 17 g/L (0.29 M) NaCl	TG was calibrated using a a non-slice selective 90 pulse
17	GE 3T Discovery MR750	custom 13C head coil			
18	GE 3T Discovery MR750w	TX 13C clamshell<br>RX 1H/13C endorectal coil (GE)	1H body coil,<br>1H/13C endorectal coil (GE)		
19	GE 3T Discovery MR750	eight-channel 13C breast coil (RAPID)	1H body coil + dedicated eight-channel phased array receive-only breast coil	13C-labeled 8 M urea	13C-labeled 8 M urea sample (Sigma-Aldrich), positioned adjacent to the tumor-containing breast, was used to set the 13C transmit gain and center frequency
20	GE 3T Discovery MR750	TX volume coil, RX 8 CH or 32 CH			
21	GE 3T Discovery MR750				
22	Siemens 3T Tim Trio	TX 2-channel transmit, RX 8-channel surface-receive array (RAPID)	6 channel flexible 1H receive array	13C urea phantom	A [13C]urea fiducial marker strapped on top of the coil was used to calibrate the 13C center frequency
23	GE 3T Discovery MR750	TX + RX custom surface coil with figure-eight configuration	16-channel abdominal array (GE Healthcare)		
24	GE 3T Discovery MR750	eight-channel 13C breast coil (RAPID)	before 13C measurement<br>8-channel RX only breast coil	8 M 13C urea phantom	

25	GE 3T Discovery MR750	TX 13C clamshell (GE)<br>RX Two 4-channel 13C surface coils arrays (GE)	TX 1H body coil<br>RX 4-channel paddle receive coil	13C urea phantom	Initial pre-scan frequency and power calibration were performed on 13C urea phantom attached outside the receive coil, which was removed before pyruvate injection. Real-time 13C frequency and power calibration and triggered upon bolus arrival.
26	GE 3T Discovery MR750	TX 13C clamshell (GE)<br>RX 1H/13C endorectal coil (GE)			
27	GE 3T Discovery MR750				
28	GE 3T Discovery MR750	TX birdcage RX 24-channel array (RAPID) or TX 13C clamshell (GE), RX Two 4-channel 13C surface coils arrays (GE)	body coil		Immediately following imaging, a non-localized spectrum was acquired to confirm center frequency, for heart studies Integrated bolus tracking, center frequency calibration, and B1+ calibration were performed
29	GE 3T Discovery MR750w	Nested-design 1H/13C: quadrature 13C TX, and 8-channel 13C RX array (Clinical MR Solutions)	Nested-design 1H/13C: quadrature 1H TX/RX (Clinical MR Solutions)	ethylene glycol (used w/ small animal coil)	
30	GE 3T Discovery MR750w	TX Helmholtz<br>RX 8-channel receive array	body coil		
31	GE 3T Discovery MR750w	Nested-design 1H/13C: quadrature 13C TX, and 8-channel 13C RX array (Clinical MR Solutions)	Nested-design 1H/13C: quadrature 1H TX/RX (Clinical MR Solutions)	Gd-doped 0.4-M spherical [13C]HCO_3– phantom (diameter = 18 cm)	single-voxel 1H point-resolved spectroscopy (PRESS) shimming up to 1st order, 13C transmit power pre-calibrated with bicarbonate phantom
32	GE 3T Discovery MR750w	Nested-design 1H/13C: quadrature 13C TX, and 8-channel 13C RX array (Clinical MR Solutions)	Nested-design 1H/13C: quadrature 1H TX/RX (Clinical MR Solutions)		
33	GE 3T Discovery MR750	8-ch 13C RX / clamshell TX, 32-ch 13C RX / Volume TX		A head-shaped phantom containing unenriched ethylene glycol doped with 17 g/L (0.29 M) NaCl, and 8M 13C-urea phantom	transmit power calibrated on a head-shaped phantom, frequency calibration with urea phantom
34	GE 3T Discovery MR750	home-built single-tuned TX/RX 13C birdcage coil using same support base as 1H coil	1H body coil;<br>8-channel neurovascular receive array (Invivo)		
35	Siemens 3T Tim Trio	TX 2 channel, RX 8 channel surface-receive array (RAPID)	1H body coil		
36	GE 3T Discovery MR750w	2-loop coil (20 cm diameter) TX/RX (PulseTeq)<br>flexible quadrature TX and 8 channel RX coil (Clinical MR Solutions)	1H body coil	saline-loaded dimethyl silicone	13C CF from 1H CF; B1+ estimated using dimethyl silicone phantoms prior to volunteer imaging
37	GE 3T Discovery MR750	TX clamshell, RX 8-ch paddle (GE)			real-time CF and B1+ calibration
38	GE 3T Discovery MR750w	2-loop coil (20 cm diameter) TX/RX (PulseTeq)			13C CF from 1H CF

39	GE 3T Discovery MR750w	Nested-design 1H/13C: quadrature 13C TX, and 8-channel 13C RX array (Clinical MR Solutions)	Nested-design 1H/13C: quadrature 1H TX/RX (Clinical MR Solutions)		
40	GE 3T Discovery MR750	single-tuned quadrature birdcage coil as a transceiver	32-channel head coil	urea	CF and B1+ from urea next to subject
41	GE 3T Discovery MR750	birdcage TX + 32-channel custom-built receiver (UCSF-MGH)/ 24-channel RX (RAPID) OR custom-built volume TX/RX birdcage coil (UCSF-MGH)		head-shaped ethylene glycol	B1+ on ethylene glycol phantom prior to subject
42	GE 3T Discovery MR750	TX/RX custom figure-8 surface coil	commercial 32-channel torso array or a 16-channel flex array	urea integrated in 13C coil; 25-cm diameter cylindrical ethylene glycol phantom	B1+ on urea + B1 correction using B1 map from ethylene glycol phantom
43	GE 3T Discovery MR750	8-channel 13C breast coil (RAPID)	1H body coil for 13C session part; 8ch RX breast array	8-M urea	
44	GE 3T Discovery MR750	8-channel 1H/24-channel 13C phased array RX with an 8-rung low-pass 13C volume TX coil (RAPID)			13C CF from 1H CF
45	GE 3T Discovery MR750w	2-loop coil (20 cm diameter) TX/RX (PulseTeq)	1H body coil		
46	GE 3T Discovery MR750	TX clamshell, RX 8-ch paddle (RAPID)	32-channel cardiac array coil (GE) after respositioning		
47	GE 3T Discovery MR750	1H/13C endorectal RX coil (RAPID)	1H/13C endorectal RX coil (RAPID)		
48	GE 3T Discovery MR750				
49	GE 3T Discovery MR750	dual-tuned 1H/13C quadrature TX/RX head coil (RAPID)	dual-tuned 1H/13C quadrature TX/RX head coil (RAPID)	8 M 13C urea attached to the ear defenders worn by the subject	13C transmit gain (TG) and center frequency were set using a13C enriched urea phantom
50	GE 3T Discovery MR750w	Nested-design 1H/13C: quadrature 13C TX, and 8-channel 13C RX array (Clinical MR Solutions)	Nested-design 1H/13C: quadrature 1H TX/RX (Clinical MR Solutions)	0.4-M bicarbonate sphere	13C CF from 1H CF; B1+ from separate phantom scan
51	GE 3T Discovery MR750	TX/RX volume head coil (UCSF/MIT or PulseTeq); volume TX + 24-channel RX (RAPID)		gadolinium-doped natural abundance dimethyl silicone 16-cm sphere (for B1+ mapping)	13C CF from 1H CF
52	GE 3T Discovery MR750	TX/RX Helmholtz loop-pair 13C coil (PulseTeq) or TX clamshell + 16-channel RX array (RAPID)	8-channel cardiac RX array and/or body coil	bicarbonate sphere	B1+ with Bloch-Siegert on bicarbpmate sphere close to patient; 13C CF from 1H CF
53	GE 3T Discovery MR750	1H/13C dual-tuned birdcage TX/RX coil (PulseTeq)	1H/13C dual-tuned birdcage TX/RX coil (PulseTeq)	Natural abundance glycerol head phantom	B1+ with a 90◦ hard pulse on glycerol phantom (presumably prior to patient); 13C CF from 1H CF
54	GE 3T Discovery MR750	1H/13C TX/RX head coil (RAPID)	12-channel head coil (GE)		

55	GE 3T Discovery MR750	dual-tuned 1H/13C quadrature TX/RX head coil (RAPID)	dual-tuned 1H/13C quadrature TX/RX head coil (RAPID)		high order shimming; Bloch-Siegert for B1+ and CF
56	GE 3T Discovery MR750	TX 13C clamshell + RX 1H/13C endorectal coil (GE)	1H body coil for TX	urea phantom inside endorectoal coil	B1+ calibration on urea phantom; real-time CF calibration
57	GE 3T Discovery MR750	fabricated flexible 8-ch 13C RX array + TX 13C clamshell (RAPID)	1H body coil for TX	natural abundance ethylene glycol (for phantom experiments)	13C CF from 1H CF; B1+ with Bloch-Siegert but not clear what signal source (23Na?)
58	GE 3T Discovery MR750	1H/13C endorectal RX coil (RAPID)	1H/13C endorectal RX coil (RAPID)		
59	Siemens 3T Biograph mMR	TX clamshell + 1H/13C endorectal RX coil (RAPID)		8M urea phantom inside endorectoal coil	13C CF and B1+ calibration on urea phantom
60	GE 3T Discovery MR750	8 ch1H/24 ch 13C phased array receiver with an 8-rung low-pass 13C volume transmit coil (RAPID)	assuming 1H body coil TX		
61	GE 3T Discovery MR750	TX 13C clamshell + 1H/13C endorectal coil (GE)	1H body coil for TX + 4ch torso array (combined with endo coil)	urea phantom inside endorectoal coil	13C CF and B1+ calibration on urea phantom
62	GE 3T Discovery MR750	flexible quadrature TX and 8 channel RX coil (Clinical MR Solutions)	1H body coil	27.8 cm-diameter spherical dimethyl silicon phantom for B1+ mapping	built-in autoshimming (in subset manual linear shim in ROI); B1+ from separate phantom scan (90deg hard pulse); 13C CF from 1H CF
63	GE 3T Discovery MR750w	volume excitation TX coil (clamshell?) + 8-channel paddle RX (GE)		head-shaped phantom containing natural abundance ethylene glycol (for phantom experiments)	

Supporting Table S2: Acquisition methods reported in HP ^{13}C-pyruvate human study papers surveyed. Note that some papers reported multiple acquisition methods. The entries are blank when the methods were not reported in the manuscript or supplementary/supporting materials. If some values were not reported, they are denoted by a "?". In some cases, the methods were ambiguous and have been inferred from the cited paper or its references, which are *italicized* and followed by "?". "~" indicates a range of parameter values were reported. N/A = Not applicable.
For the Start Time, some studies reported a single value, but it was not clear whether this was relative to the start of injection, end of injection, or other time, and these are shown as a single value and are *italicized*.
Abbreviations: EPSI = Echo-planar spectroscopic imaging; SPSP = spectral-spatial; EPI = Echo-planar imaging; IDEAL = iterative decomposition with echo asymmetry and least-squares estimation; CSI = chemical shift imaging; bSSFP = balanced steady-state free-precession; RR = time interval between heart beats

Ref	Acquisition Method (Categorical)	Acquisition Methods	Spatial resolution	Coverage	Start Time [sec]	Temporal Resolution [sec or RR intervals]	Acquired time points (start : interval : end)	Anatomical Target
1	MRS/I	1D EPSI	10 mm	18 cm x 36-60 mm slice	Start of injection+0	3	3 s	Prostate
	MRS/I	2D EPSI (dynamic)	10 mm	8 cm x 18 cm x 12-40 mm slice	Start of injection+5	5	5 s	Prostate
	MRS/I	2D EPSI (single timepoint)	7mm	8.4 cm x 8.4 cm x 10 - 20 mm slice	Start of injection+25~33	N/A	12 s	Prostate
	MRS/I	3D EPSI (single timepoint)	7 mm x 7 mm x 7-15mm	8.4 cm x 12.6 cm x 12 cm	Start of injection+25~33	N/A	8 - 12 s	Prostate
2	Metabolite-specific Imaging	2D multi-slice spiral, SPSP excitation (dynamic)	8.8 mm x 8.8 mm x 10 mm	6 cm in slice direction	End of injection	3 RR	Acquired 3 time points over 18 cardiac cycles	Heart
3	MRS/I	*EPSI?*						Prostate
4	Metabolite-specific Imaging	3D EPI	15mm x 15mm x 15mm	72cm x 72xm x 72cm	End of injection+10	6	10s after saline flush : 6s : 60s	Abdomen/Pelvis
5	MRS/I	2D EPSI (dynamic)	10-12 mm x 10-12 mm x 15-20 mm	16-20 cm x 16-20 cm x 15-20 mm slice	End of injection+0	4.3	4.3 s	Brain
6	MRS/I	Slab dynamic 2D EPSI (dynamic)	3 cm slice<br>15-20 mm x 15-20 mm x 20-30 mm slice	N/A<br>18-20 cm x 27-36 cm x 2-3 cm slice	End of injection+5	3	5s after end of injection : 3 s : end of injection + 5s+ 39*3s	Brain
7	MRS/I	3D EPSI (dynamic)	8 mm x 8 mm x 8 mm		*End of injection+5?*	2	? : 2 s : ?<br>36-s window<br>" starting 5s after injection"	Prostate
8	MRS/I	3D EPSI (dynamic)	8mm x 8mm x 8mm	9.6cm x 9.6xm x 12.8cm	End of injection+5	2	5s after saline flush : 2s : 42s<br>(21 timepoints)	Prostate
9	Metabolite-specific Imaging	EPI SPSP excitation (dynamic)				2		Prostate
9	MRS/I	EPSI (dynamic)						Prostate

10	Metabolite-specific Imaging	2D EPI multi-slice (dynamic)	8 mm x 8 mm x 8 mm<br>15 mm x 15 mm x 20 mm	12.8 cm x 12.8 cm x 12.8 cm<br>24 cm x 24 cm x 16 cm	End of injection+5	2	5 s after end of injection: 2 s : end of injection+5s+20*2s	Methods
11	MRS/I	2D EPSI (dynamic)	1.8cm x 1.8cm x 2cm	? x 28.8cm x 2cm		3	? : 3s : 60s (20 timepoints)	Abdomen/Pelvis
	Metabolite-specific Imaging	2D EPI (dynamic)	1.5 x 1.5cm x 2cm	24cm x 24cm x 16cm		3	? : 3s : 60s (20 timepoints)	Brain
12	Chemical Shift Encoding	2D IDEAL spiral , multi-slice (dynamic)	12 mm x 12 mm x 30 mm	24 cm x 24 cm x 3 cm	End of injection+10	4	10s after end of injection : 4 s : ?	Brain
13	MRS/I	2D CSI (dynamic)	7.5 mm x 7.5 mm x 30 mm	12 cm x 12 cm x 3 cm slice	*25*	20	25 s : 20 s : 245 s	Kidney
14	Metabolite-specific Imaging	2D EPI multi-slice, SPSP excitation (dynamic)	15 mm x 15 mm x 20 mm slice	? x ? x 16 cm slice	End of injection+5	3	5s after end of injection : 3 s : ?	Brain
15	MRS/I	2D CSI (dynamic)	16 mm x 16 mm x 30 mm	16 cm x 16 cm x 3 cm	*25*	10	25 s : 10 s : 120 s	Breast
16	Metabolite-specific Imaging	2D EPI multi-slice, SPSP excitation (dynamic)	2-8 cm^3	24 cm x 24 cm x variable	End of injection+5	3	5s after end of flush : 3 s : 60 s<br>(20 time points)	Brain
17	Metabolite-specific Imaging	3D EPI (dynamic, dual-echo)	15 mm x 15 mm x 15 mm	24 cm x 24 cm x 36 cm	End of injection+0	5	End of injection: 5 s : end of injection + 60s	Brain
18	MRS/I	2D EPSI (dynamic)	10 mm x 10 mm x 15 mm	16 cm x 16 cm x 15 mm slice	Start of injection+5	4.9	4.9 s	Prostate
19	Chemical Shift Encoding	2D IDEAL spiral (2-3 slices)	Reconstructed to 2 mm x 2mm x 30 mm slice		*12 or End of injection+12?*	4	*? "delay = 12 s"*	Breast
20	Metabolite-specific Imaging	2D EPI multi-slice (dynamic)	12-15 mm x 12-15 mm x 15-20 mm	? x ? x 12-16 cm slice	End of injection+5	3	5s after end of injection: 3s : end of injection+5s+39*3s	Brain
21	MRS/I	Slab dynamic		NA	End of injection+0	1	25 s : 1 s : 55 s	Pancreas
	MRS/I	2D CSI (single time point)	15 mm x 15 mm x 20 mm	24 cm x 24 cm x 20 mm slice	*35*	N/A	35s	Pancreas
22	MRS/I	pulse-acquire	10 mm slice	Coil FOV x 10 mm slice	*0*	1 RR	0s : 1 RR interval : up to 240 s	Heart
23	MRS/I	2D EPSI (dynamic)	1.2-1.5cm x 1.2-1.5cm x 2-3cm	? x ? x 2-3cm	Start of injection+5	3	? : 3s : 60s	Abdomen/Pelvis
24	Chemical Shift Encoding	IDEAL spiral						Breast
25	Metabolite-specific Imaging	Lactate imaging: 3D bSSFP, stack of spirals (dynamic)<br>Pyruvate/alanine	15 mm x 15 mm x 21 mm	69 cm x 69 cm x 33.6 cm<br>45 cm x 45 cm x 33.6 cm	Detected bolus arrival+6	3.5	6s after bolus arrival : 3.5 s : ?	Kidney

		imaging: 2D GRE multi-slice, spiral readout (dynamic)						
26	Metabolite-specific Imaging	2D EPI multi-slice, SPSP excitation (dynamic)	8 mm x 8 mm x 8 mm slice	12.8 cm x 12.8 cm x 11.2 cm		2	?: 2 s : ?	Prostate
27	MRS/I	2D EPSI (dynamic)	1.2-2cm x 1.2-2cm x 1.5-4cm slice			2~5	? : 2-5s : (20-24 time points)	Methods
28	Metabolite-specific Imaging	2D EPI multi-slice, SPSP excitation (dynamic)	7.5 mm x 7.5 mm x 15 mm slice (pyruvate)<br>15 mm x 15 mm x 15 mm slice (lactate and bicarbonate)	24 cm x 24 cm x 12 cm	End of injection+5	3	5s after end of flush : 3 s : 60 s (20 time points)	Brain
	Metabolite-specific Imaging	2D spiral Gated multi-slice, SPSP excitation (dynamic)		2.1 cm slice x 5 slices		3 RR		Heart
29	MRS/I	2D spiral CSI single-slice (dynamic)	15 mm x 15 mm x 2-3 cm slice	24 cm x 24 cm x 2-3 cm	*3*	5	? : 5 s : ?	Brain
30	MRS/I	Slab spectra (dynamic)	10-cm slab	10-cm slab		2.8-3.8 (*3 RR?*)	? : 2.8-3.8 s : 3.7-5 min (80 time points)	Heart
31	MRS/I	2D spiral CSI single-slice (dynamic)	15 mm x 15 mm x 30 mm	24 cm x 24 cm x 3 cm	*5*	5	? : 5 s : ?	Brain
32	MRS/I	Spiral CSI (dynamic)	15 mm x 15 mm x 25-30 mm	24 cm x 24 cm x 2.5-3 cm	*5*	5	5s after start of injection : 5 s : 90 s	Brain
33	Metabolite-specific Imaging	2D EPI multi-slice, SPSP excitation (dynamic)	3.38-4.5 cm^3	24 cm x 24 cm x 8 slices	End of injection+0~5	3	0-5 s after end of flush: 3 s : 60 s (20 time points)	Brain
	MRS/I	EPSI (dynamic)	8 cm^3		End of injection+0~5	3	0-5 s after end of flush: 3 s : 72 s (24 time points	Brain
34	Metabolite-specific Imaging	3D EPI with SPSP excitation (dynamic)	15 mm x 15 mm x 15 mm	24 cm x 24 cm x 36 cm	*0*	5	0 s : 5 s : 60 s	Brain
35	Metabolite-specific Imaging	2D hybrid-shot spiral multi-slice, SPSP excitation	10 mm x 10 mm x 20 mm<br><br>6 mm x 6 mm x 10 mm	? x ? x 60 mm<br><br>38.4 cm x 38.4 cm x 12 cm	End of injection+22		Data acquired over 9 heartbeats, starting 22 s after injection<br>Data acquired over 36 heartbeats, starting 22 s after injection	Heart
36	Metabolite-specific Imaging	2D spiral, SPSP excitation (dynamic)	6.1 mm x 6.1 mm x ?	40 cm x 40 cm x ?	End of injection+0	6 RR	end of injection: 6 RR : 50 s	Heart

37	Metabolite-specific Imaging	2D EPI multi-slice, SPSP excitation (dynamic)	15 mm x 15 mm x 21 mm		Detected bolus arrival+6	4	6s after bolus arrival : 4s : ?	Kidney
	Metabolite-specific Imaging	3D bSSFP (Lactate only)	15 mm x 15 mm x 21 mm		Detected bolus arrival+6	4	6s after bolus arrival : 4s : ?	Kidney
38	Metabolite-specific Imaging	2D spiral multi-slice, SPSP excitation (dynamic)	16 mm x 16 mm x 30 mm	40 cm x 40 cm x 30 mm	*25*	2 RR	25s after start of injection : 2 RR : 25s + 16 timepoints.	Heart
39	MRS/I	Slab spectra (dynamic)	100 mm slab	100 mm slab		3	? : 3 s : 240 s	Muscle
	Metabolite-specific Imaging	Multi-echo spiral with metabolite-specific SPSP excitation	? x ? x 80 mm slice thickness		*33*	N/A		Muscle
40	Metabolite-specific Imaging	2D EPI multi-slice, SPSP excitation (dynamic)	15 mm x 15 mm x 15 mm	? x ? x 12 cm	End of injection	3	0s : 3s : 57s	Brain
41	Metabolite-specific Imaging	2D EPI multi-slice, SPSP excitation (dynamic)	15 mm x 15 mm x 15 mm	24 cm x 24 cm x 12 cm	End of injection+5	3	0 : 3 s : 60 s	Brain
42	MRS/I	2D EPSI with multi-band SPSP excitation (dynamic)	12 mm x 12 mm x 12-30 mm	? x ? x 12-30 mm	End of injection+5	3	0 : 3 s : 60 s	Liver
43	Metabolite-specific Imaging	Metabolite-specific SPSP excitation and spiral readout. One patient- IDEAL spiral	5 mm x 5 mm x ?	20 cm x 20 cm x ?		4	? : 4 s : ?	Breast
	Chemical Shift Encoding	Metabolite-specific SPSP excitation and spiral readout. One patient- IDEAL spiral	5 mm x 5 mm x ?	20 cm x 20 cm x ?		4	? : 4 s : ?	Breast
44	Metabolite-specific Imaging	Metabolite specific EPI with SPSP excitation (dynamic)	15 mm x 15 mm x 15 mm	24 cm x 24 cm x 12 cm	Start of injection+5	3	0 s (5s after end of flush): 3 s : 60 s	Brain
45	Metabolite-specific Imaging	Metabolite-specific SPSP excitation and spiral readout (dynamic)	10 mm x 10 mm x 30 mm	40 cm x 40 cm x 3 cm	*25*	1 RR	Started at 25s after start of injection. One acquisition per RR interval.	Heart

46	Chemical Shift Encoding	IDEAL spiral multi-slice (dynamic)	17 mm x 17 mm x 30 mm	34 cm x 34 cm x 17 cm (5 slices with 5 mm slice gap)	*12*	4	? : 0.5 s (?) : ? "starting 12 s after the injection of HP-pyruvate"	Kidney
47	Chemical Shift Encoding	IDEAL spiral (dynamic)	12.5 mm x 12.5 mm x ?	20 cm x 20 cm x ?		4	0 s : 4 s : 80 s	Prostate
48								Prostate
49	Chemical Shift Encoding	IDEAL spiral	6 mm x 6 mm x 30 mm	24 cm x 24 cm x 3 cm	*10*	4	10 s after injection : 4 s : 70 s	Brain
50	MRS/I	Slab MRS (dynamic)	15 mm slab	15 mm slab		3	? : 3 s : 240 s	Brain
51	Metabolite-specific Imaging	EPI with SPSP excitation (dynamic)	7.5-15 mm x 7.5-15 mm x 15 mm		End of injection+2	3	2 s after end of injection: 3 s : ?	Brain
52	Metabolite-specific Imaging	Metabolite-specific SPSP with spiral readout (dynamic)	13.3 mm x 13.3 mm x 30 mm	40 cm x 40 cm x 3 cm		3 RR	Acquired 40 images of each metabolite with a time resolution of 3 heart beats (images in diastole)	Heart
53	Metabolite-specific Imaging	Metabolite-specific SPSP with spiral readout (dynamic)	0.875 mm x 0.875 mm x 20 mm (pyruvate). 1.75 mm x 1.75 mm x 20 mm (lactate/bicarbonate)	? x ? x 12 cm	End of injection+0	2	: 2s :	Brain
54	Chemical Shift Encoding	IDEAL spiral	6 mm x 6 mm x 30 mm	24 cm x 24 cm x 3 cm		4	0 s : 4 s : 60 s	Brain
55	MRS/I	2D CSI multi-slice (single time point)	20 mm x 20 mm x 20 mm	20 cm x 20 cm x 10 cm	*22~27*	N/A	22-27 s "after injection"	Brain
56	Metabolite-specific Imaging	Metabolite-specific 2D GRE (pyruvate/lactate) Metabolite-specific 3D-bSSFP (urea) (dynamic)	7 mm x 7 mm x 11.6 mm	Pyruvate/lactate: 22.4 cm x 22.4 cm x 11.6 cm Urea: 21.7 cm x 21.7 cm x 18.56 cm	End of injection+8	2.6	8s after end of flush : 2.6 s : 52 s	Prostate
57	Metabolite-specific Imaging	Metabolite-specific stack of spirals (dynamic)	7 x 7 x 15 mm (pyruvate) 14x14x15 mm (lactate/alanine/bicarbonate)	28 x 28 x 12 cm	Start of injection+0	1.232	0 s after end of pyruvate injection, before flush : 1.232 : ?	Brain
58	Chemical Shift Encoding					4		Prostate
59	Chemical Shift Encoding	multi-echo bSSFP (dynamic)	11.3 mm x 11.3 mm x 10 mm	90 mm x 90 mm x 80 mm	Start of injection+0	6	0 s after end of injection: 6 s : 204 s	Prostate

60	Metabolite-specific Imaging	2D EPI with SPSP excitation multi-slice (Dynamic)	7.5 mm x 7.5 mm x 15-20 mm (pyruvate). 15mm x 15 mm x 15-20 mm (lactate/bicarbonate)	24 cm x 24 cm x 1.5-2 cm (pyruvate). 48 cm x 48 cm x 1.5-2cm (lactate/bicarbonate)	End of injection+0~5	3	0 s (5s after end of flush OR immediately after end of flush) : 3s : 60s	Brain
61	Metabolite-specific Imaging	Metabolite-specific EPI with SPSP excitation (dynamic)	6.5-8mm x 6.5-8mm x 8 mm	10.4-12.8 cm x 10.4-12.8 cm x 11.2 cm	Start of injection+10		0 s (10s after end of flush):	Prostate
62	MRS/I	2D EPSI (dynamic)	20-22 mm x 20-22 mm 20-30 mm	32-35.2cm x 36-39.6cm x 2-3cm	End of injection+5	3	5s after end of flush: 3 s : 60 s	Liver
	Metabolite-specific Imaging	EPI (dynamic)	20 mm x 20 mm x 20 mm	32 cm x 32 cm x 2 cm	End of injection+5	3	5s after end of flush: 3 s : 60 s	Liver
63	MRS/I	2D EPSI (dynamic)	12.5 mm x 12.5 mm x 15 mm	20 cm x 20 cm x 1.5 cm	Start of injection+0	4.3	0s: 4.3 s: 58 timepoints	Methods

Supporting Table S3: Metrics reported in HP ^{13}C-pyruvate human study papers surveyed.

Abbreviations: pyr = pyruvate; lac = lactate; ala = alanine; bic = bicarbonate; AUC = area-under-curve; tC = total Carbon-13 signal; PK = pharmacokinetic; kPL, kPA, kPB, kLP are kinetic rates; ve and vb are voxel extracellular and vascular fractions, respectively; T1eff = T1 effective; TTP = time to peak; NAWM = normal appearing white matter; NAB = normal appearing brain

Khegai PK model reference: Khegai O, Schulte RF, Janich MA, et al. Apparent rate constant mapping using hyperpolarized [1-(13)C]pyruvate. NMR Biomed. 2014;27(10):1256-1265. doi:10.1002/nbm.3174

Inputless PK model reference: Larson PEZ, Chen HY, Gordon JW, et al. Investigation of analysis methods for hyperpolarized 13C-pyruvate metabolic MRI in prostate cancer patients. NMR Biomed. 2018;31(11):e3997. doi:10.1002/nbm.3997; Hyperpolarized-MRI-Toolbox. https://github.com/LarsonLab/hyperpolarized-mri-toolbox, doi:10.5281/zenodo.1198915

Reference	Metrics	Values Reported	Model Parameters
1	kPL mean<br>lac/pyr	0.009 – 0.013 sec-1	Fit T1pyr, T1lac<br>No reverse conv
2	AUC Pyr<br>AUC Lac<br>AUC Bic	~95-140 (plot)<br>~35-65<br>~30-80	
3	kPL max (Prostate tumor)	0.007 - .025 sec-1	model not specified
4	AUC pyr<br>AUC lac<br>AUC ala<br>AUC Bic	~50-13500 (colorbar)<br>~50-920<br>~50-670<br>~50-550	
5	AUC pyr<br>AUC lac<br>TTP pyr<br>TTP lac<br>kPL, full 2-comp model	~0.0-0.7<br>~0.0-0.1<br>11.7 +/- 1.9<br>23.0 +/- 1.3<br>0.12 (0.08-0.16) s-1	Fixed T1pyr;<br>Constrained T1lac, ve
6	SNR lac max (normal)<br>SNR bic max (normal)<br>SNR lac max (tumor)<br>SNR bic max (tumor)<br>Lac/pyr (normal)<br>Bic/pyr (normal)<br>Lac/pyr (tumor)<br>Bic/pyr (tumor)	28.0-79.1<br>9.2-34.1<br>4.0-51.6<br>1.5-5.8<br>0.18-0.98<br>0.07-0.37<br>0.30-0.58<br>0.02-0.08	
7	kPL<br>SNR pyr (last, mean)<br>SNR lac (last, mean)<br>SNR total 13C<br>SNR total 13C (normal tissue)	~.005-.0.020 sec-1 (colorbar)<br>104, 45.2<br>10.7, 6.1<br>51.3<br>48.2	Fit kLP=0; Fit t1, T1s equal
8	Inputless kPL max<br>Inputless kPL mean<br>AUC lac/AUC pyr<br>kPL with input<br>TTP Lac<br>Mean lac time<br>Mean pyr time<br>SNR total Pyr, max<br>SNR total Lac, max	0.023 (0.009 - 0.049)<br>0.009 (0.003-0.018)<br><br><br><br><br>29.0 (24.8-32.3) sec<br>628.8 (189.2-1415.0)<br>99.4 (49.5-195.5)	Fit T1pyr, T1LAC; kLP=0<br>Fixed T1lac; kLP=0<br>Fixed T1lac; kLP=0<br>From PK model w/input<br>Fixed T1LAC, fit input

9	kPL<br>Unconstrained<br>Constrained	 ~0.00-0.02 s-1<br>~0.001-0.0075 s-1	Fitting with vascular input function(defined)<br>kLP=vb=0;
10	Dynamic images<br>AUC pyr, lac, bic (brain)<br>Peak SNR pyr, lac, bic (brain)<br>TTP pyr, lac, bic (brain)	 0-1, 0-0.28, 0-0.07<br>396-447, 16-30, 6-8<br>3-9, 6-12, 9-15	
11	Pyr AUC (brain)<br>Lac AUC (brain)<br>Bic AUC (brain)<br>Lac/Pyr AUC ratio (brain)<br>Bic/Pyr AUC ratio (brain)<br>Lac/Pyr (liver tumor)<br>Lac/Pyr (normal liver)	~0-0.75<br>~0-0.22<br>~0-0.12<br>~0-1<br>~0-0.5<br>0.200-0.306<br>0.070-0.108	
12	AUC: pyr (norm to max pyr)<br>Lac (norm to max pyr)<br>Bic (norm to max pyr)<br>AUC ratios: lac/pyr<br>Bic/pyr<br>Bic/lac<br>kPL<br>kPB	 <br><br>0.18±0.04 - 0.25±0.08<br>0.04±0.02 - 0.08±0.05<br>0.20±0.02 – 0.33±0.1<br>0.008 - 0.024 s-1<br>0.002 – 0.003 s-1	
13	Pyr<br>lac<br>Lac/pyr	~5-35<br>~1-5.5<br>~0.1-0.55	 First timepoint (20s)
14	Pyr SNR<br>Lac SNR<br>Bic SNR		
15	Pyr (t=25s)<br>Lac (t=25s)	~3-16<br>~0.5-2.5	
16	kPL (NAWM)<br>kPB (NAWM)<br>kPL (lesion)<br>kPL-lesion/kPL-NAWM	0.014-0.047 s-1<br>0.0033-0.011 s-1<br>0.012-0.51<br>0.76-1.30	“inputless” PK model
17	Lac AUC<br>Bic AUC<br>Lac Z-Score<br>Bic Z-Score	Image<br>Image<br>~-3-2.6<br>~-2.3-3	Z-score as the standard deviation from average
18	Pyr TTP<br>Lac TTP<br>Max Lac/tC<br>Cumulative normalized Lac	11.2-33.5 s<br>~20-61s<br>0.1485-0.6383<br>0.0885-0.4200	
19	Lac/Pyr AUC ratio<br>Total Pyr SNR<br>Total Lac SNR<br>kPL	~0-0.6<br>~10-160<br>~0-50<br>~0-0.45 s-1	Two-way PK; equal T1s; vb=0
20	kPL,<br>kLP,<br>kPB	0.009-0.05<br><br>~0-0.009	(models defined)<br>Fit T1 assuming equal for metabolites; vb=0

	Mean arrival time<br>Max SNR pyr, lac, bic	6-11s<br>101-437,13-79,11-27	
21	Lac/Pyr<br>Ala/Pyr<br>Ala/Lac	0.36<br>0.12<br>0.33	
22	Bic/Pyr AUC ratio<br>Lac/Pyr AUC ratio<br>Bic/Lac AUC ratio<br>Ala/Pyr AUC ratio	0.0016-0.0184<br>0.0516-0.1053<br>0.02-0.30<br>0.0317-0.0474	
23	kPL<br>SNR tC	0.013-0.026 s-1<br>19.5-290.3	“inputless” PK model
24	kPL<br>Lac/Pyr AUC ratio	~0.15-0.25<br>~0.30-0.50	Khegai model
25	Lac/Pyr AUC ratio<br>AUC Images	~0-2.4	
26	kPL’	~0.005-0.035	2 physical compartment model with arterial input function<br>T1Pyr, T1Lac assumed
27	Metabolite images<br>SNR<br>kPL	 <br>0.0261-0.0369 s-1	“inputless” PK model
28	Pyr SNR<br>Lac SNR<br>Bic SNR<br>Peak Pyr SNR<br>Peak Lac SNR<br>Peak Bic SNR<br>Pyr SNR (left ventricle)<br>Lac SNR<br>Bic SNR<br>Pyr AUC<br>Lac AUC<br>Bic AUC	~0-87<br>~0-15<br>~0-6<br>288.8<br>14.8<br>6.3<br>~0-906<br>~0-50<br>~0-14<br>~1.6-162<br>~0.3-30<br>~0.1-4.0	
29	Pyr Signal Intensity<br>Lac SI	Images,<br>Dynamic Curves	
30	Bic/tC<br>Lac/tC<br>Ala/tC	0.032-0.038<br>0.061-0.448<br>0.045-0.051	
31	Bic/tC<br>Lac/tC	0.025-0.059<br>0.162-0.236	
32	Lac/tC AUC (Tumor)<br>Lac/tC AUC (NAB)<br>Bic/tC AUC (Tumor)<br>Bic/tC AUC (NAB)	0.160-0.327<br>0.148-0.268<br>0.062-0.111<br>0.077-0.124	
33	Pyr AUC<br>Lac AUC<br>Bic AUC<br>Lac/Pyr AUC Ratio<br>Pyr AUC SNR (EPI)<br>Lac AUC SNR (EPI)<br>Bic AUC SNR (EPI)	~0-88<br>~0-76<br>~0-26<br>~0-1.8<br>785-1070<br>175-243<br>64-83	

	Pyr AUC SNR (EPSI)<br>Lac AUC SNR (EPSI)<br>Bic AUC SNR (EPSI)	4750-352000<br>522-72000<br>304-15200	
34	Lac Z-score	-7	
35	Pyr AUC<br>Lac AUC<br>Bic AUC	~10-80<br>~2-12<br>~1-6	
36	Bic SNR<br>Pyr AUC<br>Lac AUC<br>Bic AUC	6.8 – 14.9<br>Figure<br>Figure<br>Figure	
37	Pyr SNR<br>Lac SNR<br>Ala SNR<br>Pyr AUC<br>Lac AUC<br>Ala AUC<br>Lac/pyr AUC ratio<br>Ala/pyr AUC ratio	75 ± 49<br>13 ± 5.8<br>4.2 ± 2.0<br>Figure<br>Figure<br>Figure<br>~0.5 – 1.6<br>0.48 – 1.72	
38	Pyr T2*<br>Lac T2*<br>Bic T2*<br>Pyr SNR<br>Lac SNR<br>Bic SNR	109 – 129 ms<br>41 – 44 ms<br>64 ms<br>~100 – 250<br>~10 – 50<br>~10 – 100	
39	Total carbon AUC<br>Pyr/tC AUC ratio<br>Lac/tC AUC ratio<br>Ala/tC AUC ratio<br>Bic/tC AUC ratio<br>Ala/lac AUC ratio<br>Bic/lac AUC ratio	 0.41 – 0.66<br>0.18 – 0.42<br>0.11 – 0.22<br>0.002 – 0.004<br>0.28 – 0.72<br>0.004 – 0.036	tC adjusted for polarization
40	kPL<br>Lac/Pyr AUC ratio<br>Pyr relative cerebral blood flow (rCBF)<br>Pyr mean transit time (MTT)	~0.10-0.30<br>~0.2-0.75<br>~0.5-2.5<br>~12.5-25 s	'Inputless' PK model
41	kPL<br>kPB<br>Peak Pyr AUC SNR<br>Peak Lac AUC SNR<br>Peak Bic AUC SNR	Mean:0.0133-0.0221 s-1<br>Mean:0.0022-0.0093 s-1<br>160-393<br>17-56<br>7-13	Model not specified; T1s fit
42	Pyr SNR fold-increase<br>Lac SNR fold-increase<br>Ala SNR fold-increase<br>kPL	 <br><br>0.027 – 0.073 s-1	'Inputless' PK model
43	Pyr SNR<br>Lac SNR<br>Lac/pyr AUC ratio<br>kPL	16.5 – 19.7<br>5.6 – 7<br>0.28 – 0.34<br>0.0064 – 0.0079 s-1	PK model not specified (SVD recon)
43	Pyr SNR<br>Lac SNR	16.5 – 19.7<br>5.6 – 7	PK model not specified (SVD recon)

	Lac/pyr AUC ratio<br>kPL	0.28 – 0.34<br>0.0064 – 0.0079 s-1	
44	*Global*<br>Pyr SNR fold-increase with denoising<br>Lac SNR fold-increase with denoising<br>Bic SNR fold-increase with denoising<br><br>*NAWM*<br>kPL<br>kPB<br>Bic/lac AUC ratio<br><br>*Non-enhancing lesion:*<br>kPL<br>kPB<br>Bic/lac AUC ratio<br><br>*Contrast enhancing lesion*<br>kPL<br>kPB<br>Bic/lac AUC ratio	 3.9 +/- 0.8 (NOT SNR..)<br>3.1 +/- 1.1<br>5.1 +/- 2.2<br><br><br>~0.015 – ~0.033 s-1<br>~0.0035 – ~0.005 s-1<br>~0.15 – ~0.275<br><br><br>~0.01 – ~0.04 s-1<br>~0.0025 s-1<br>~0.1 – ~0.15<br><br><br>~0.0175 – ~0.02 s-1<br>~0.002 s-1<br>~0.075 – ~0.1	 'inputless' PK model
45	Bic/tC AUC ratio<br>Lac/tC AUC ratio<br>Ala/tC AUC ratio<br>Bic/lac AUC ratio<br>Bic /Ala AUC ratio	0.04 – 0.22<br>0.143 – 0.17<br>0.07 – 0.13<br>0.3 – 1.4<br>0.63 – 2.6	
46	*Tumor*<br>Pyr SNR<br>Lac SNR<br>Lac/pyr AUC ratio<br>kPL<br><br>*Contralateral*<br>Pyr SNR<br>Lac SNR<br>Lac/pyr AUC ratio<br>kPL	 26.7 (16.8-61.3)<br>5.7 (1.9-9.6)<br>0.13 – 0.35<br>0.0022 – 0.0152 s-1<br><br><br>30.1 (14.8-64-7)<br>3.5 (2-9.3)<br>0.14<br>0.0043 s-1	PK with Khegai model. Two-site exchange with T 1eff
47	*Tumor*<br>Total carbon SNR<br>Pyr SNR<br>Lac SNR<br>kPL	 27 – 112<br>15 – 70<br>6 – 40<br>0.003 – 0.018 s-1	PK with Khegai model. Two-site exchange with T 1eff
48	kPL	0 – 0.027 s-1	Not reported
49	Lac AUC<br>Pyr AUC<br>Lac/(lac+pyr) AUC ratio	 <br>~0.21	
50	Lac/tC<br>Bic/tC<br>Ala/tC<br>Bic/lac<br>Pyr-hydrate/pyr	0.21 – 0.24<br>0.065 – 0-091<br>0.002 – 0.026<br>0.3 – 0.38<br>0.048 – 0-067	Ratios from time-averaged spectra<br><br>Three-site exchange PK model, fitting of T1's and k's.

	Lac/pyr<br>Bic/pyr<br>Pyr-hydrate/tC<br><br>Upslope lac / upslope pyr<br>Upslope bic / upslope pyr<br>TTP pyr<br>TTP lac<br>TTP bic<br><br>kPL<br>kPB	~0.38<br>~0.12<br>~0.04<br><br>~0.06<br>~0.015<br>~17 s<br>~27 s<br>~34 s<br><br>~0.015<br>~0.005	
51	Pyr SNR<br>Lac SNR<br><br>kPL<br>Lac/pyr AUC ratio<br>Lac Z-score	10.9<br>13.8<br><br>~0.015 – 0.025 s-1<br>~0.2 – 0.67	‘Inputless’ PK model
52	Pyr SNR<br>Lac SNR<br>Ala SNR<br>Bic SNR<br>Lac TTP<br>Ala TTP<br>Bic TTP<br>Pyr TTP<br>Pyr first order moment<br><br>kPL<br>kPB<br>kPA	150 – 290<br>7 – 19<br>9 – 17<br>7 – 12<br>13 – 16 s<br>6 – 11 s<br>13 – 17 s<br>6 – 3 s<br>7 - 17.8 s<br><br>0.011 – 0.02 s-1<br>0.004 – 0.012 s-1<br>0.005 – 0.016 s-1	‘Inputless’ PK model
53	kPL<br>kPB<br>Lac/bic AUC ratio<br>Pyr mean transit time	0.014 – 0.023 s-1<br>~0.0065 s-1<br>0.4 – 0.045<br>~15 s	‘Inputless’ PK model
54	*Normal brain*<br>Pyr AUC / peak pyr<br>Lac AUC / peak pyr<br>Bic AUC / peak pyr<br>Lac/pyr AUC ratio<br>Bic/pyr AUC ratio<br>kPL<br>kPB<br><br>*Glioblastoma multiforme*<br>Pyr AUC / peak pyr<br>Lac AUC / peak pyr<br>Bic AUC / peak pyr<br>Lac/pyr AUC ratio<br>Bic/pyr AUC ratio	 0.14 – 0.24<br>0.04 – 0.09<br>0.009 – 0.027<br>0.36 +/-0.06<br>0.1 +/- 0.03<br>0.0165 +/- 0.0073 s-1<br>0.0024 +/- 0.001 s-1<br><br><br>0.19 – 0.41<br>0.05 – 0.13<br>0.006 – 0.022<br>0.34 +/- 0.06<br>0.06 +/- 0.03	AUC ratios noise-corrected<br>PK with Khegai model. Two-site exchange with T 1eff

	kPL<br>kPB	0.0161 +/- 0.0057 s-1<br>0.0017 +/- 0.0013 s-1	
55	Pyr/max tC<br>Lac/max tC<br>Bic/max tC<br>Lac/pyr<br>Bic/pyr<br>Lac/bic	0.63 ± 0.09<br>0.28 ± 0.09<br>0.08 ± 0.02<br>0.48 ± 0.23<br>0.13 ± 0.03<br>3.68 ± 1.21	
56	Pyr AUC<br>Lac AUC<br>Lac/pyr AUC ratio	~30-150<br>~10-25<br>~0.1-0.5	
57			
58	*Tumor*<br>Total carbon SNR<br>Pyr SNR<br>Lac SNR<br>kPL	 42.7 – 94.1<br>22.6 – 61.2<br>9.2 – 23.1<br>0.005 – 0.018 s-1	PK with Khegai model. Two-site exchange with T 1eff
59	lac/pyr AUC ratio<br><br>kPL	0.33 +/- 0/12 (tumor), 0.15+/- 0.10 (healthy)<br>0.038 /- 0.014 s-1 (tumor), 0.011 +/- 0.007 s-1 (healthy)	unidirectional PK model, T1pyr and kPL estimated
60	Lac/pyr AUC ratio<br>kPL	0.24 – 0.42<br>0.01 – 0.015 s-1	'Inputless' PK model
61	kPL	0.0198 – 0.041 s-1	'Inputless' PK model
62	Pyr SNR<br>Lac SNR<br>Ala SNR<br><br>Relative pyr TTP<br><br>Pyr AUC<br>Lac AUC<br>Ala AUC<br>kPL<br>kPA	 <br><br><br><br><br><br><br><br>0.0033 – 0.019 s-1<br>0.00073 – 0.012 s-1	'Inputless' PK model
63	SNR (spatial uniformity)	Images	NA
